# α7nAChR agonist GTS‐21 ameliorates sepsis‐induced acute kidney injury via MEF2/PGC‐1α/HO‐1 axis in mice

**DOI:** 10.1002/ctm2.70726

**Published:** 2026-06-23

**Authors:** Yu‐Jia Tang, Hui‐Ying Liu, Na‐Qi Li, Xin Zhang, Yi‐Lu Lin, Yan Zhang, Yao Li, Jia‐Le Deng, Pei‐Lin Yang, Qing‐Min Meng, Yi‐Jin Tang, Zi‐Yue Zhang, Si‐Han Guan, Kai Kang, Hong‐Liang Wang, Yang Gao

**Affiliations:** ^1^ Department of Critical Care Medicine The Sixth Affiliated Hospital of Harbin Medical University Harbin Heilongjiang Province People's Republic of China; ^2^ Department of Nephrology The First Affiliated Hospital of Harbin Medical University Harbin Heilongjiang Province People's Republic of China; ^3^ Department of Critical Care Medicine The Second Affiliated Hospital of Harbin Medical University Harbin Heilongjiang Province People's Republic of China; ^4^ Department of Critical Care Medicine The Fourth Affiliated Hospital of Harbin Medical University Harbin Heilongjiang Province People's Republic of China; ^5^ Department of Critical Care Medicine The First Affiliated Hospital of Harbin Medical University Harbin Heilongjiang Province People's Republic of China

**Keywords:** α7nAChR, HO‐1, MEF2, mitochondria, PGC‐1α, sepsis‐induced acute kidney injury

## Abstract

**Background:**

Sepsis‐induced acute kidney injury (S‐AKI) is a major global public health concern, yet effective therapeutic strategies remain limited. Mitochondrial dysfunction in renal tissues is a key pathogenic mechanism underlying S‐AKI. GTS‐21, a selective α7 nicotinic acetylcholine receptor (α7nAChR) agonist, exhibits anti‐inflammatory and renoprotective effects in S‐AKI.

**Methods:**

We investigated the role of α7nAChR in S‐AKI using both in vitro (lipopolysaccharide (LPS)‐induced renal tubular cell injury) and in vivo (caecal ligation and puncture (CLP)‐induced septic mice) models, with GTS‐21 treatment.

**Results:**

GTS‐21 significantly attenuated mitochondrial dysfunction, suppressed apoptosis, and alleviated inflammation, thereby protecting renal tubular cells and renal tissues against LPS‐ and CLP‐induced injury. Mechanistically, GTS‐21 activated α7nAChR and upregulated myocyte enhancer factor 2 (MEF2), peroxisome proliferator‐activated receptor gamma coactivator 1‐alpha (PGC‐1α), and heme oxygenase‐1 (HO‐1), which collectively mediate its anti‐oxidative, anti‐apoptotic and anti‐inflammatory effects.

**Conclusion:**

These findings suggest that GTS‐21 may represent a potential therapeutic strategy for sepsis‐induced kidney injury.

## Key points

- GTS‐21 alleviates sepsis‐associated acute kidney injury by preserving mitochondrial function and suppressing apoptosis.
- Activation of α7nAChR promotes MEF2‐dependent upregulation of PGC‐1α and HO‐1.
- The α7nAChR/MEF2/PGC‐1α/HO‐1 signalling axis represents a potential therapeutic target for sepsis‐associated acute kidney injury.

## Graphical abstract

This diagram illustrates opposing pathways regulating AKI (acute kidney injury). The left pathway shows LPS/TLR4 triggering mitochondrial damage (low MMP, Cyt C release, ROS) leading to apoptosis. The right pathway shows α7nAChR agonists activating MEF2 to upregulate PGC‐1α/HO‐1, promoting mitochondrial biogenesis and inhibiting injury, highlighting neural‐immune regulation.

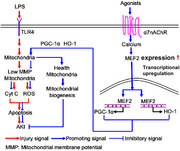

## 1 INTRODUCTION

Sepsis is a life‐threatening systemic inflammatory syndrome characterized by multiple organ dysfunction syndrome (MODS) resulting from a dysregulated host response to infection.^1^ Acute kidney injury (AKI) is a frequent and severe complication in sepsis, often manifesting as acute tubular necrosis (ATN) and contributing substantially to poor clinical outcomes.^2^, ^3^ Epidemiological studies indicate that sepsis accounts for approximately 20% of global mortality annually, while AKI occurs in nearly 30%–50% of septic patients, significantly worsening prognosis.^4^, ^5^ Despite extensive research efforts, the current management of sepsis‐induced AKI (S‐AKI) remains largely supportive, and no targeted therapies have demonstrated consistent clinical efficacy.^6^ These sobering realities underscore the urgent need to elucidate novel pathogenic mechanisms and develop effective targeted therapeutic strategies to improve outcomes in S‐AKI.

Mitochondrial dysfunction has been increasingly recognized as a central contributor to the pathophysiology of S‐AKI.^6^ Sepsis‐associated systemic inflammation and endotoxemia significantly impair mitochondrial oxidative phosphorylation, leading to reduced adenosine triphosphate (ATP) production and subsequent apoptosis and/or necrosis of renal tubular epithelial cells due to energy deficiency.^2^ In sepsis, mitochondrial dysfunction not only promotes excessive reactive oxygen species (ROS) production but also exacerbates oxidative stress and inflammatory responses, thereby establishing a vicious cycle that ultimately disrupts cellular homeostasis and leads to organ dysfunction.^7^ In addition, dysregulated mitochondrial dynamics, characterized by excessive fission and impaired autophagy, further aggravate cellular injury.^8^ Under conditions of cellular stress, mitochondria undergo swelling and outer membrane permeabilization, resulting in the release of cytochrome c (CytC) into the cytosol. Cyt c then interacts with apoptotic peptidase activating factor 1 (Apaf‐1), forming the apoptosome and initiating caspase‐dependent apoptotic cell death.^9^ Accumulating evidence has demonstrated increased expression of oxidative stress‐related genes, substantial mitochondrial DNA damage, and markedly reduced mitochondrial mass in both patients and experimental models of S‐AKI.^8^, ^10^ In contrast, enhanced mitochondrial biogenesis has been associated with recovery of mitochondrial integrity and renal function in AKI models.^11^ Therapeutic strategies aimed at restoring mitochondrial structure and ATP production have shown potential to reduce renal tubular epithelial cell apoptosis and partially preserve renal function in ischemia–reperfusion and AKI models.^12^ Collectively, these findings highlight that restoration of mitochondrial function and biogenesis represents a critical therapeutic target for promoting renal recovery in AKI.

The α7 nicotinic acetylcholine receptor (α7nAChR), a homopentameric ligand‐gated ion channel, is widely expressed in both the nervous system and non‐neuronal tissues, including neurons, immune cells and renal tubular epithelial cells.^13^, ^14^, ^15^ As a key component of the cholinergic anti‐inflammatory pathway, α7nAChR activation robustly modulates systemic inflammatory responses by suppression of pro‐inflammatory cytokine release.^16^ Emerging evidence suggests that α7nAChR not only regulates immune responses but also exerts cytoprotective effects in multiple disease models through the modulation of mitochondrial function. In experimental models of neurodegenerative diseases and ischemia/reperfusion injury, pharmacological activation of α7nAChR using selective agonists such as GTS‐21 has been shown to significantly attenuate tissue injury by restoring mitochondrial membrane potential (MMP) and reducing reactive oxygen species (ROS) production.^17^, ^18^ In sepsis‐associated pathologies, α7nAChR activation has consistently been reported to alleviate systemic inflammation and organ dysfunction. For instance, GTS‐21 administration attenuates atrial inflammation in septic mice by inhibiting pro‐inflammatory macrophage polarization, reducing oxidative stress restoring mitochondrial function in cardiomyocytes.^19^ However, whether α7nAChR agonists confer protection against S‐AKI through direct regulation of mitochondrial function in renal tubular epithelial cells remains unclear. Therefore, systematic investigation of α7nAChR‐mediated mitochondrial regulation in renal tubular epithelial cells may provide novel therapeutic insights for the treatment of S‐AKI.

Mitochondrial biogenesis is orchestrated by multiple transcription factors, including myocyte enhancer factor 2 (MEF2), peroxisome proliferator‐activated receptors (PPARs) and estrogen‐related receptors (ERRs).^20^, ^21^ These signalling pathways converge on the master regulator of mitochondrial biogenesis, peroxisome proliferator‐activated receptor gamma coactivator‐1 alpha (PGC‐1α). PGC‐1α serves as a central regulator of mitochondrial biogenesis, reactive oxygen species (ROS) homeostasis and autophagic flux. In addition, PGC‐1α directly regulates the expression of multiple antioxidant defence genes, including glutathione peroxidases, catalase and mitochondria‐localized peroxidases, thereby coordinating cellular antioxidant defence systems.^22^ Heme oxygenase‐1 (HO‐1) is a stress‐inducible antioxidant enzyme that catalyses the rate‐limiting step in heme degradation, generating carbon monoxide (CO), ferrous iron (Fe^2^^+^) and biliverdin, which is subsequently converted to bilirubin.^23^ Notably, HO‐1 and its metabolic products (CO, Fe^2^^+^ and biliverdin/bilirubin) exert potent antioxidant and cytoprotective effects.^24^ In addition, HO‐1 displays strong anti‐inflammatory activity, largely mediated through CO‐dependent modulation of pro‐ and anti‐inflammatory cytokine signalling pathways.^25^ Collectively, these findings define an integrated transcriptional network that links mitochondrial biogenesis with coordinated antioxidant and anti‐inflammatory defence systems. Recent studies have shown that α7nAChR activation in glial cells promotes mitochondrial biogenesis and enhances cellular bioenergetics in a manner dependent on HO‐1‐ and PGC‐1α‐associated signalling pathways.^15^ However, the precise molecular mechanisms by which α7nAChR regulates mitochondrial function in S‐AKI remain incompletely understood.

The present study was designed to elucidate the mechanisms by which α7nAChR agonists ameliorate S‐AKI. To recapitulate the pathological features of S‐AKI, we employed complementary in vitro and in vivo models, including LPS‐challenged renal tubular epithelial cells and CLP‐induced septic mice. Our data demonstrate that α7nAChR agonisted treatment effectively attenuates LPS‐ and CLP‐induced mitochondrial dysfunction. Mechanistically, α7nAChR activation upregulates myocyte enhancer MEF2, PGC‐1α and HO‐1, thereby promoting mitochondrial biogenesis and ultimately alleviating S‐AKI pathogenesis. Collectively, these findings provide mechanistic insights into α7nAChR‐mediated renal protection and identify the MEF2/PGC‐1α/HO‐1 axis as a potential therapeutic target for S‐AKI intervention.

## 2 METHODS AND MATERIALS

### 2.1 Primary mouse renal tubular epithelial cells (mRTECs) culture and identification

Kidney tissues were harvested from anaesthetized mice and washed with PBS. The tissues were minced into small fragments using sterile scissors and digested with 0.25% trypsin (containing 0.01% DNase I) at 37°C for 20 min. The digestion was terminated by adding DMEM supplemented with 10% foetal bovine serum (FBS). After filtration through a 200‐mesh sieve, the cell suspension was centrifuged at 1500 rpm for 5 min, and the supernatant was discarded. The resulting pellet was further digested with 0.1 % Type I collagenase at 37°C for 20 min. Following termination of digestion, cells were resuspended in complete DMEM in sterile 1.5 mL centrifuge tubes and subsequently seeded into 10‐cm culture dishes for attachment and expansion. Cells were maintained at 37°C in a humidified incubator with 5% CO_2_. After 48–72 h of culture, cells were detached with 0.25% trypsin and seeded into a 12‐well plate at a density of 1 × 10^5^ cells per well. Cells were then immunofluorescently stained with an epithelial cell marker CK18 primary antibody (Proteintech, Cat#10830‐1‐AP, 1:500) to determine the presence of epithelial cells. All in vitro experiments were performed using a conventional two‐dimensional (2D) monolayer culture system.

### 2.2 HK‐2 cells culture

HK‐2 cells (human renal proximal tubular epithelial cells) were cultured in DMEM supplemented with 10 % FBS and penicillin (100 IU/mL)/streptomycin (100 µg/mL) at 37°C in a humidified incubator with 5% CO_2_.

### 2.3 Cell treatment

HK‐2 cells and mRTECs were seeded into 96‐well plates at a density of 5 × 10^3^ cells per well. Upon reaching approximately 80% confluence, cells were treated with GTS‐21 (GTS), dexmedetomidine (DEX), PNU‐282987 (PUN), AR‐R17779 (AR) and SSR180711 (SSR) at concentrations of 33 nM, 100 nM, 300 nM, 900 nM, 2.7 µM and 8.1 µM for 24 h to determine the optimal concentration for subsequent experiments. The concentrations used in subsequent experiments were selected based on the highest concentrations that did not significantly inhibit cell proliferation, thereby minimizing cytotoxic effects while allowing sufficient evaluation of pharmacological efficacy. Detailed dose‐screening results are provided in the Supporting Information.

To establish an in vitro sepsis model, HK‐2 cells and mRTECs were stimulated with LPS (1 µg/mL). Cells were seeded into 6‐well plates at a density of 5 × 10^5^ cells per well. Upon reaching approximately 80% confluence, cells were treated with GTS‐21 or the indicated compounds for 24 h. The experimental groups were as follows: control, LPS (1 µg/mL), LPS + GTS‐21 (2.7 µM), LPS + DEX (33 nM), LPS + PNU (900 nM), LPS + AR (33 nM) and LPS + SSR (900 nM). Subsequent analyses were performed after 24 h of treatment.

### 2.4 Cell transfection

HK‐2 cells were seeded into 6‐well plates at a density of 5 × 10^5^ cells per well. Upon reaching approximately 80% confluence, cells were transfected with α7nAChR siRNA (GCGAGTTCCAGAGGAAGCTTTACAA, General Biosystems Corp. Ltd.) using TransIntro EL Transfection Reagent (TransGen Biotech, FT201) according to the manufacturer's instructions. Cells were harvested 48 h post‐transfection for subsequent experiments. In addition, α7nAChR knockout (KO) mRTECs were isolated from genetically modified mice.

For MEF2 knockdown, sh‐MEF2 lentivirus (Applied Biological Materials) was transduced into HK‐2 cells and wild‐type mRTECs. Briefly, when cells reached approximately 80% confluence, lentivirus was added and incubated for 24 h, followed by replacement with fresh complete medium. After 48 h, transduced cells were selected using puromycin (1 µg/mL).

### 2.5 S‐AKI animal model construction

Wild‐type and α7nAChR knockout C57BL/6 mice (6–8 weeks old) were obtained from Aniphe Biolaboratory Inc. All animal procedures were performed in accordance with the Guide for the Care and Use of Laboratory Animals (National Institutes of Health) and approved by the Institutional Animal Care and Use Committee of The Sixth Affiliated Hospital of Harbin Medical University. (Approval No. LC2024‐058).

The CLP model was established as previously described.^26^ Briefly, mice were anaesthetized using 3% isoflurane (Sigma, 792632) and the abdominal area was shaved and disinfected. A midline laparotomy was performed to expose the caecum, which was ligated distal to the ileocecal valve using a 4‐0 silk suture. The ligated caecum was then punctured twice with a sterile needle, and a small amount of faecal material was gently extruded. The caecum was returned to the abdominal cavity, and the incision was closed in layers. Sham‐operated mice underwent the same procedure without ligation or puncture.

For survival analysis, six mice were included in each group and monitored daily for 7 days. Survival rates were recorded. At 24 h post‐surgery, mice were euthanized with an overdose of phenobarbital (100 mg/kg).

### 2.6 Drug administration and adeno‐associated Virus (AAV) infection

Mice in the CLP + GTS group received GTS‐21 (4 mg/kg; MCE, HY‐14564A) via intraperitoneal injection 12 h after CLP surgery.

For gene modulation, sh‐MEF2 AAV (Applied Biological Materials) was administered via tail vein injection to generate MEF2‐deficient mice. In addition, the PGC‐1α inhibitor SR‐18292 (MCE, HY‐101491) and the HO‐1 inhibitor tin protoporphyrin IX (MCE, HY‐101194) were dissolved in DMSO and administered via intraperitoneal injection at a dose of 5 mg/kg at 12 h post‐CLP.

### 2.7 Cell viability assay

Cell viability was assessed using a Cell Counting Kit‐8 (CCK‐8) assay (Beyotime, C0039) according to the manufacturer's instructions.

### 2.8 MMP assessment

MMP was evaluated using a JC‐1 Mitochondrial Membrane Potential assay kit (YEASEN, 40705ES08). In normal mitochondria, JC‐1 forms aggregates that emit red fluorescence, whereas in depolarized mitochondria, JC‐1 remains in the monomeric form and emits green fluorescence. HK‐2 cells and mRTECs were collected, incubated with JC‐1 according to the manufacturer's protocol, and analysed by flow cytometry (NovoCyte Penteon, Agilent Technologies).

For in vivo analysis, kidney tissues were harvested from mice and processed into single‐cell suspensions. Briefly, approximately 100 mg of kidney tissue was excised, minced into small fragments and digested with collagenase I and 0.25% trypsin at 37°C for 1 h. The digestion was terminated by adding complete medium, followed by filtration through a 200‐mesh cell strainer. The resulting cell suspension was centrifuged at 1000 rpm for 5 min, and the pellet was resuspended in PBS. MMP was then assessed using the JC‐1 assay as described above.

### 2.9 Mitochondrial ROS detection

Mitochondrial ROS levels were measured using MitoSOX Red (YEASEN, 40778ES50) in both cells and kidney tissue‐derived single‐cell suspensions. Samples were prepared as described above and incubated with the working solution at 37°C for 30 min in the dark, according to the manufacturer's instructions. After washing three times with pre‐warmed PBS, mitochondrial ROS levels were analysed by flow cytometry.

### 2.10 Annexin V apoptosis detection assay

HK‐2 cells and mRTECs were collected, washed once with PBS, and resuspended in 195 µL Annexin V‐FITC binding buffer. Subsequently, 5 µL Annexin V‐FITC (YEASEN, 40302ES50) and 10 µL propidium iodide (PI) staining solution (YEASEN, 40302ES50) were added, followed by incubation at room temperature in the dark for 15 min. Apoptosis was analysed by flow cytometry. For in vivo experiments, single‐cell suspensions prepared as described above were subjected to the same apoptosis assay.

### 2.11 Transmission electron microscopy (TEM)

Kidney tissues and cells were collected and fixed in 2.5% glutaraldehyde for 24 h, followed by washing with PBS. Samples were then post‐fixed in 1% osmium tetroxide for 2 h, dehydrated through a graded ethanol series, embedded in epoxy resin, and sectioned into ultrathin slices. Sections were stained with uranyl acetate and lead citrate and examined using a transmission electron microscope (JEM1400, JEOL Ltd.).

### 2.12 Real‐time PCR

Total RNA was extracted from tissues or cells using TRIzol reagent (YEASEN, 19202ES60). cDNA was synthesized using a reverse transcription kit according to the manufacturer's instructions. Quantitative real‐time PCR was performed using SYBR Green Master Mix (YEASEN, 11200ES50) on a real‐time PCR system (ABI 7500, ThermoFisher Scientific).

The amplification conditions were as follows: an initial denaturation at 95°C for 5 min, followed by 40 cycles of 95°C for 10 s and 60°C for 30 s. Relative gene expression levels were normalized to GAPDH and calculated using the 2^−ΔΔCt^ method. Primer sequences are listed in Table 1.

**TABLE 1:** The primer sequences of mRNAs.

Gene Name	Primers (5’–3’)
Homo‐MEF2‐F	TCAACCTCTTGCTACCCCAGTC
Homo‐MEF2‐R	GCTTGTCCTAGGTGGTGCTGCT
Homo‐HO‐1‐F	GCCTCCCTGTACCACATCTATG
Homo‐HO‐1‐R	CTGGTGTGTAGGGGATGACCTC
Homo‐PGC‐1α‐F	CTGACCACAAACGATGACCCTC
Homo‐PGC‐1α‐R	TCTTGGTTGGCTTTATGAGGAG
Homo‐GAPDH‐F	CGGAGTCAACGGATTTGGTCGTAT
Homo‐GAPDH‐R	AGCCTTCTCCATGGTGGTGAAGAC
Mus‐MEF2‐F	CACCTACATAACATGCCGCCA
Mus‐MEF2‐R	TCGCTCCCATCGTAGGAACTG
Mus‐HO‐1‐F	GCCTCACTGGCAGGAAATCATC
Mus‐HO‐1‐R	ACCCAGGTAGCGGGTATATGCG
Mus‐PGC‐1α‐F	ATACACAACCGCAGTCGCAAC
Mus‐PGC‐1α‐R	CAAGAGGGCTTCAGCTTTGGC
Mus‐GAPDH‐F	GCCAAAAGGGTCATCATCTCC
Mus‐GAPDH‐R	GTGATGGCATGGACTGTGGTC

### 2.13 Western blot

Tissues and cells were lysed with radio immunoprecipitation assay (RIPA) (Servicebio, G2002), which contained 1 mM phenylmethylsulfonyl fluoride (PMSF) (Beyotime, ST506). The mixture was centrifuged at 12 000 g, 4°C for 5 min to collect the protein in the supernatant. The protein concentration was detected using the BCA kit (YEASEN, 20201ES86). The protein was diluted with PBS to an equal amount and then boiled with 5× loading buffer (Beyotime, P0286) for 10 min. Proteins (50 µg) were separated by sodium dodecyl sulphate‐polyacrylamide gel electrophoresis (SDS‐PAGE) and then transferred to polyvinylidene difluoride (PVDF) membrane (MERCK, IPFL00010). After blocking with 5 % degreased milk for 1 h, membranes were incubated with specific primary antibodies overnight at 4°C. After being washed three times with TBST, the membranes were incubated for 1 h with goat anti‐mouse IgG‐HRP (YEASEN, 33201ES60, 1:10 000) and goat anti‐rabbit IgG‐HRP (YEASEN, 33101ES60, 1:10 000) for 2 h at room temperature. Membranes were treated with Enhanced Chemiluminescence (ECL) reagents (Servicebio, G2020) and visualized with (OI‐X6, GuangZhou GuangYi Biotechnology Co., Ltd.). The primary antibodies included Cytochrome c Polyclonal antibody (Proteintech, Cat#10993‐1‐AP, 1:5000), HO‐1/HMOX1 Polyclonal antibody (Proteintech, Cat#10701‐1‐AP, 1:3000), MEF2A Polyclonal antibody (Proteintech, Cat#12382‐1‐AP, 1:1000), and PGC‐1α Monoclonal antibody (Proteintech, Cat#66369‐1‐Ig, 1:10 000)

### 2.14 Luciferase reporter assay

The following plasmids were transfected into HK‐2 cells using the EL transfection reagent in the following four groups: PCMV‐MYC + pRL‐TK + pGL3‐Basic; PCMV‐MYC + pRL‐TK + pGL3‐Basic‐PGC‐1α; PCMV‐MYC‐MEF2 + pRL‐TK + pGL3‐Basic; PCMV‐MYC‐MEF2 + pRL‐TK + pGL3‐Basic‐PGC‐1α. After 48 h of incubation, dual‐luciferase reporter assays were performed following the instructions provided by the UElandy Dual‐Luciferase Reporter Assay Kit (UElandy, F6075M). Renilla luciferase and firefly luciferase activities were measured using a multifunctional microplate reader (Flexstation 3, MD).

### 2.15 Serum creatinine measurement

Blood samples were collected from mice and then centrifuged at 3000 rpm for 15 min to obtain serum. The serum was added into the biochemical reaction cuvette, and the creatinine level was measured using the Direxion CS‐T180 automated biochemical analyser (DiruiIndustrial co., Ltd., CS‐T180).

### 2.16 Haematoxylin‐eosin (HE) staining

Kidney tissues were fixed in 4% paraformaldehyde (PFA), embedded in paraffin, and then sectioned. HE staining was performed to identify pathological changes in the kidney.

### 2.17 Enzyme‐linked immunosorbent assay (ELISA)

Serum levels of inflammatory mediators (IL‐1β, TNF‐α, IL‐6, NGAL) were determined using ELISA kits (Shanghai Jianglai Bio, JL18442/JL10484/JL20268/JL11556) according to the manufacturer's instructions. Absorbance was read at 450 nm using a microplate reader (Flexstation 3, MD).

### 2.18 Statistical analysis

Data analysis and processing were performed using Image J and SPSS 26.0, with all values presented as mean ± standard deviation (SD). The comparison between two groups was conducted using an unpaired *t*‐test, and differences among multiple groups were analysed using one‐way analysis of variance (ANOVA). *p* < .05 was considered statistically significant, and *p *< .01 was considered highly significant. Data shown in different figures represent independent biological experiments and are not intended for cross‐figure comparison. Each experimental group included 3 mice (*n* = 3 per group), unless otherwise specified. The sample size was determined based on previous studies using similar CLP‐induced sepsis models.

## 3 RESULTS

### 3.1 α7nAChR agonist attenuates LPS‐induced renal tubular cytotoxicity in vitro

To evaluate the protective effects of α7nAChR agonists against LPS‐induced tubular injury, an in vitro injury model was established in HK‐2 cells and mRTECs. Multiple α7nAChR agonists, including GTS‐21 (2.7 µM), dexmedetomidine (DEX, 33 nM), PNU‐282987 (PNU, 900 nM), AR‐R17779 (AR, 33 nM), and SSR180711 (SSR, 900 nM), were initially screened to identify the most suitable agonist for subsequent mechanistic investigations. The concentrations of all agonists were determined based on preliminary CCK‐8 assays, and the highest concentration that did not cause obvious inhibition of cell viability was selected for subsequent experiments.

**FIGURE 1:**
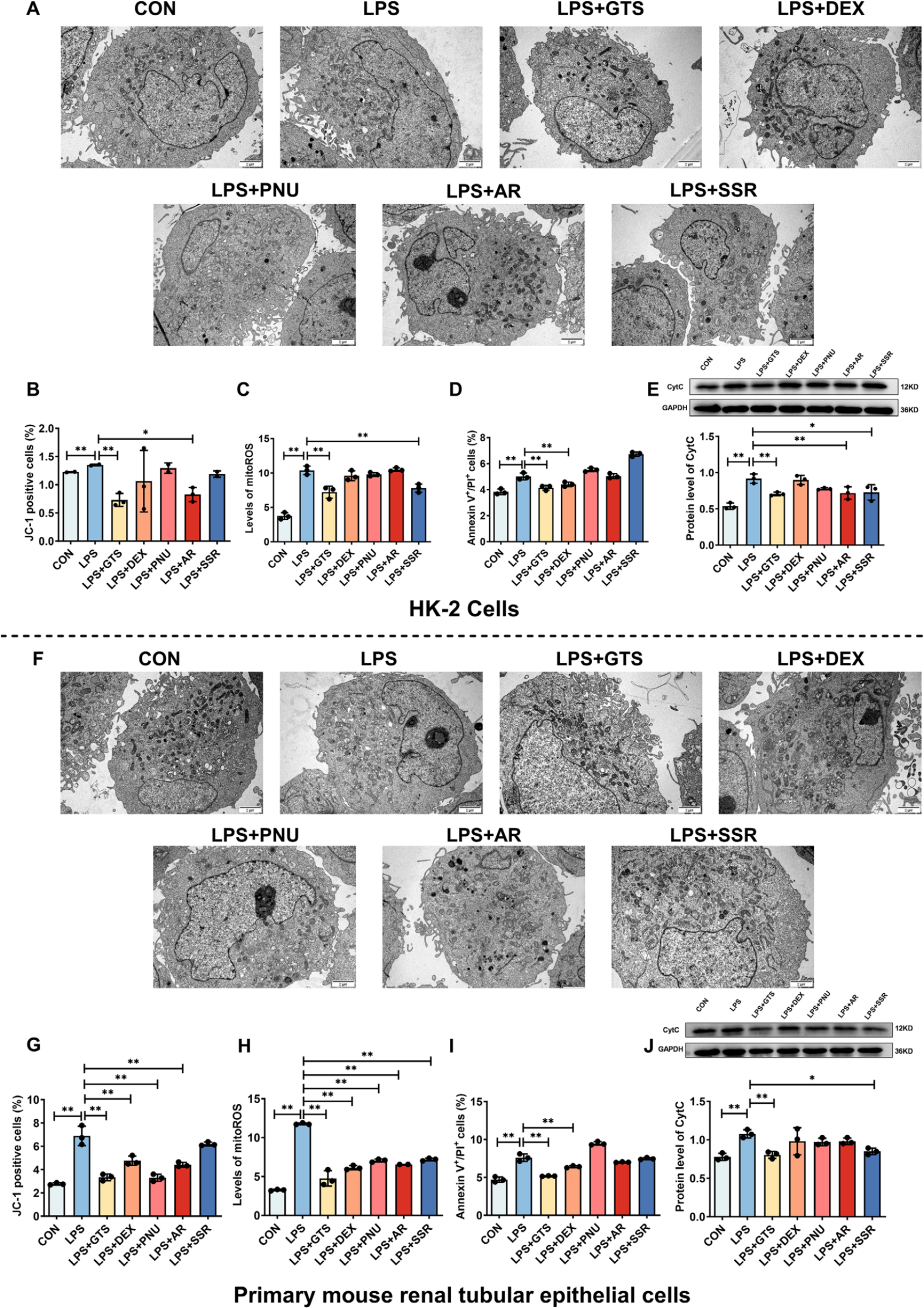
α7nAChR agonists attenuate LPS‐induced renal tubular cell injury in vitro. (A) Representative TEM images of HK‐2 cells. Scale bar = 2 µm. (B–E) Quantitative analyses of MMP, mito‐ROS levels, apoptosis, and Cyt c release in HK‐2 cells following treatment with different α7nAChR agonists. (F) Representative TEM images of mRTECs. Scale bar = 2 µm. (G–J) Quantitative analyses of MMP, mito‐ROS levels, apoptosis, and CytC release in mRTECs following treatment with different α7nAChR agonists. Data are presented as mean ± SD. *p < .05, **p < .01, ***p < .001.

TEM analysis demonstrated that control cells exhibited intact mitochondria with typical oval morphology and well‐organized cristae (Figure 1A). In contrast, LPS stimulation induced marked mitochondrial damage, characterized by mitochondrial swelling, disruption of the outer mitochondrial membrane, partial rupture and severe cristae disorganization with a significant reduction in cristae density. Treatment with α7nAChR agonists markedly alleviated these ultrastructural abnormalities in HK‐2 cells. Consistent with these observations, LPS significantly decreased mitochondrial membrane potential (MMP) and increased mitochondrial ROS levels. Among the tested agonists, GTS‐21 and AR restored MMP, whereas GTS‐21 and SSR significantly reduced mitochondrial ROS accumulation (Figure 1B,C). Furthermore, GTS‐21 and DEX markedly inhibited LPS‐induced apoptosis in HK‐2 cells (Figure 1D), while GTS‐21, AR and SSR significantly suppressed LPS‐induced cytochrome c release (Figure 1E).

To further verify the involvement of α7nAChR in the mitochondrial protective effects of GTS‐21, the selective α7nAChR antagonist methyllycaconitine (MLA) was additionally introduced. As shown in Figures S2, S3, compared with the LPS group, MLA treatment further aggravated mitochondrial dysfunction, as evidenced by decreased MMP, increased mitochondrial ROS accumulation, and enhanced apoptosis. Importantly, co‐treatment with GTS‐21 partially reversed these pathological alterations in the LPS+MLA+GTS group, resulting in restoration of MMP, reduction of mitoROS levels and attenuation of apoptotic cell death. These findings further support the important role of α7nAChR signalling in mediating the mitochondrial protective effects of GTS‐21 under inflammatory conditions.

To further validate the robustness and reproducibility of these findings, primary mRTECs were examined. As shown in Figure 1F–J, LPS stimulation induced severe mitochondrial ultrastructural disruption, decreased MMP, elevated mitochondrial ROS production, and increased apoptotic cell death in mRTECs, consistent with the observations in HK‐2 cells. Notably, treatment with α7nAChR agonists, particularly GTS‐21, markedly reversed these pathological alterations, as evidenced by improved mitochondrial structural integrity, restoration of MMP, suppression of mitochondrial ROS generation, and attenuation of apoptosis. Similar protective effects of GTS‐21 in the presence of MLA were also observed in mRTECs (Figure S3), further confirming the involvement of α7nAChR signalling in mitochondrial protection.

Collectively, although several α7nAChR agonists exhibited varying degrees of cytoprotective effects, GTS‐21 demonstrated the most stable and consistent protective effects across multiple functional endpoints and in both immortalized and primary renal tubular epithelial cells. In addition, GTS‐21 has been more extensively characterized in inflammatory and sepsis‐related disease models and possesses favourable translational potential. Therefore, GTS‐21 was selected as the representative α7nAChR agonist for subsequent mechanistic studies.

### 3.2 α7nAChR knockout reverses the protective effects of GTS‐21 against LPS‐induced renal tubular cell injury

**FIGURE 2:**
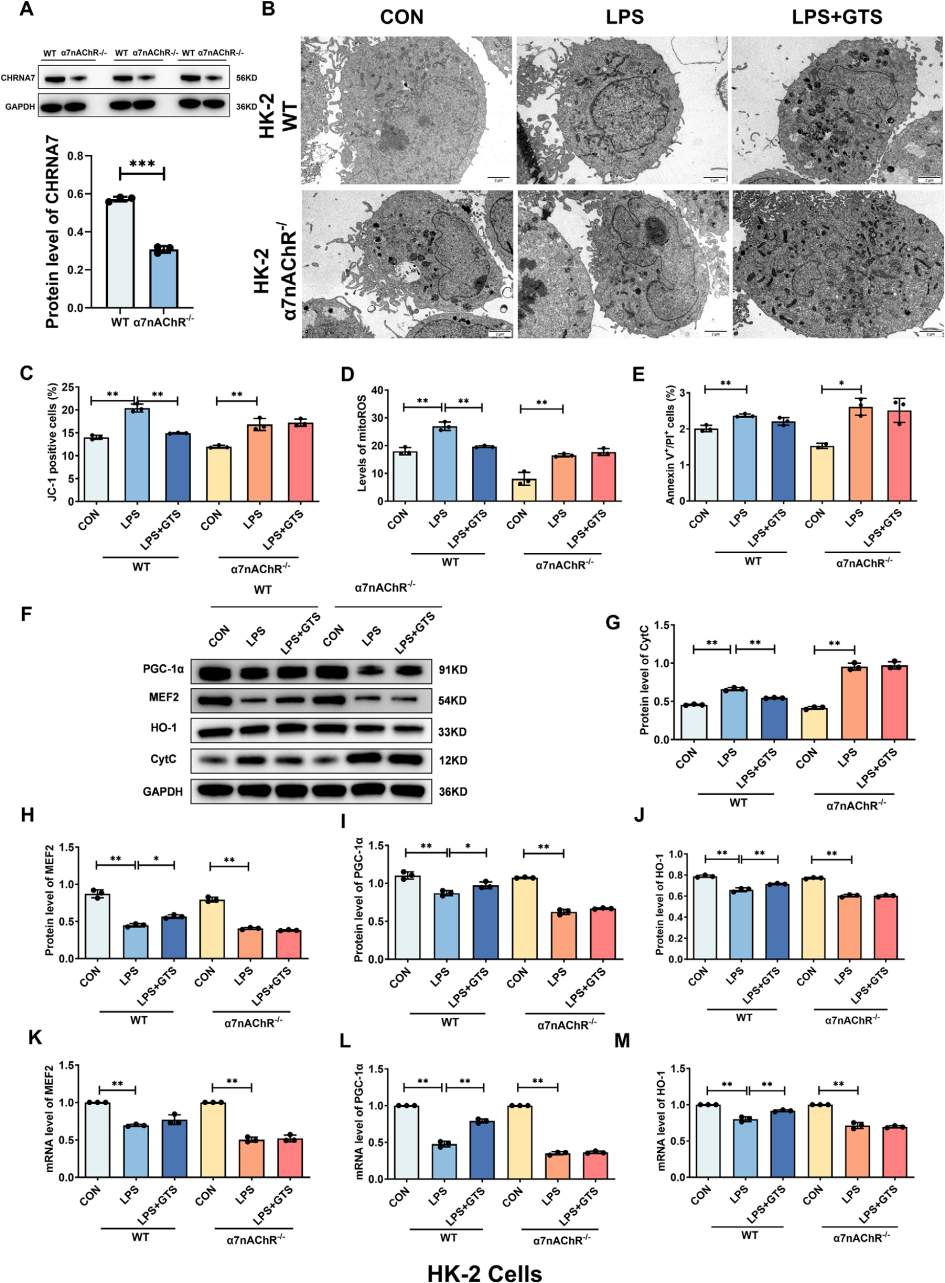
α7nAChR knockout reverses the protective effects of GTS‐21 against LPS‐induced injury in HK‐2 cells. (A) Validation of α7nAChR deficiency in HK‐2 cells. (B) Representative TEM images of HK‐2 cells. Scale bar = 2 µm. (C–E) Effects of α7nAChR on MMP, mito‐ROS levels and apoptosis in HK‐2 cells. (F–J) Representative Western blot images and quantitative analysis of CytC, MEF2, HO‐1 and PGC‐1α in HK‐2 cells. (K–M) mRNA expression levels of MEF2, HO‐1 and PGC‐1α in HK‐2 cells. Data are presented as mean ± SD. ^*^ *p* < .05, ^**^ *p* < .01.

**FIGURE 3:**
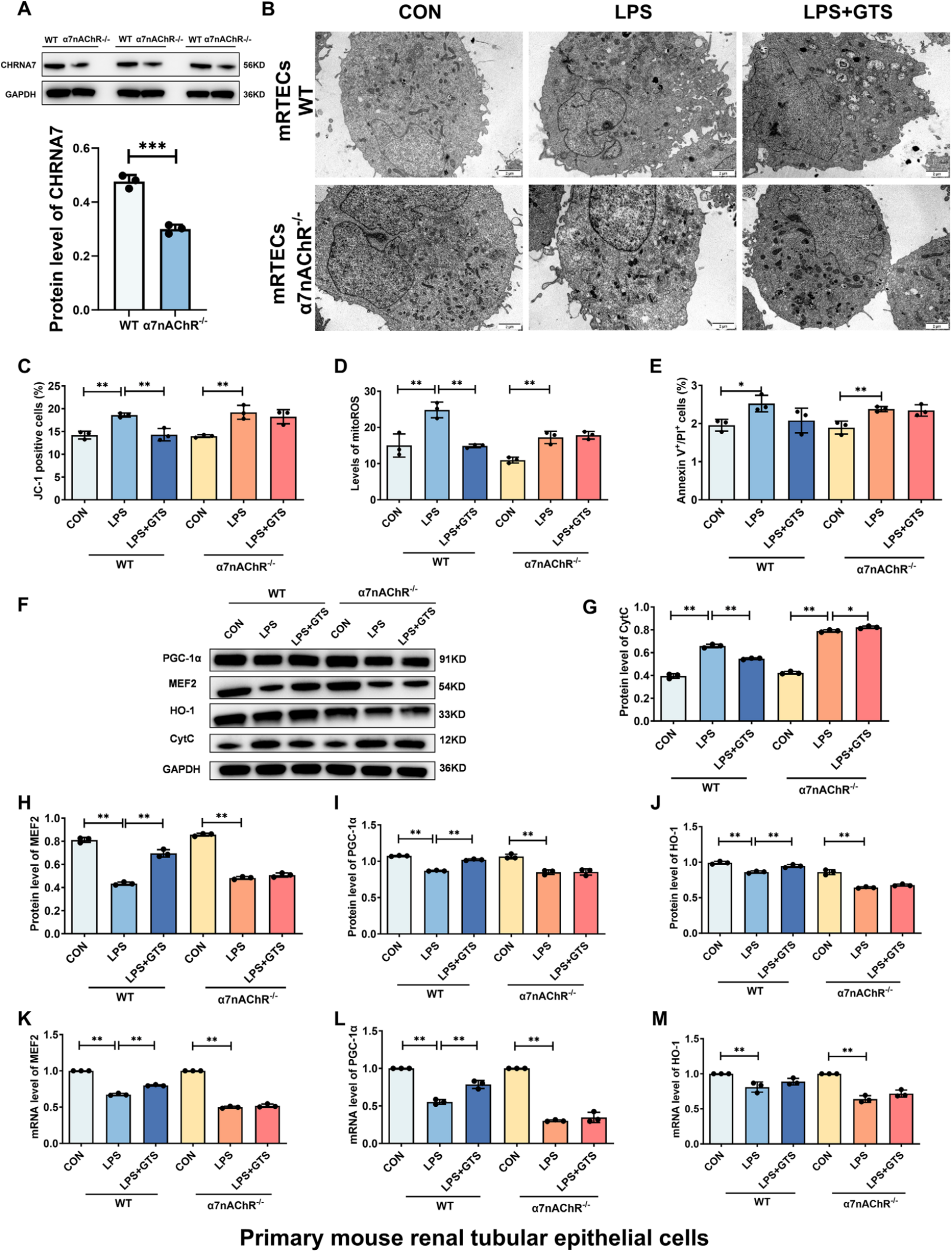
α7nAChR knockout reverses the protective effects of GTS‐21 against LPS‐induced injury in mRTECs. (A) Validation of α7nAChR deficiency in mRTECs. (B) Representative TEM images of mRTECs. Scale bar = 2 µm. (C–E) Effects of α7nAChR deficiency on MMP, mito‐ROS levels and apoptosis in mRTECs. (F–J) Representative Western blot images and quantitative analysis of CytC, MEF2, HO‐1 and PGC‐1α in mRTECs. (K–M) mRNA expression levels of MEF2, HO‐1 and PGC‐1α in mRTECs. Data are presented as mean ± SD. ^*^ *p* < .05, ^**^ *p* < .01.

To determine whether the protective effects of GTS‐21 are dependent on α7nAChR, α7nAChR‐deficient HK‐2 cells and mRTECs were generated. Successful knockdown of α7nAChR in HK‐2 cells was confirmed by Western blot analysis, which demonstrated a marked reduction in α7nAChR protein expression compared with WT cells (Figure 2A). TEM analysis showed that α7nAChR deficiency abolished the protective effects of GTS‐21 on mitochondrial ultrastructure, as evidenced by disrupted membrane integrity and reduced cristae density in HK‐2 cells (Figure 2B). Consistently, α7nAChR deficiency markedly reversed GTS‐21‐mediated restoration of MMP, suppression of mitochondrial ROS levels, inhibition of apoptosis, and reduction of Cyt c release (Figure 2C–G). These findings indicate that the protective effects of GTS‐21 are largely dependent on α7nAChR activation. Furthermore, LPS stimulation significantly decreased the expression levels of MEF2, HO‐1, and PGC‐1α in HK‐2 cells. GTS‐21 treatment restored the expression of these factors; however, this effect was abolished in α7nAChR‐deficient cells (Figure 2E, H–M). Similar results were observed in mRTECs (Figure 3). Collectively, these results demonstrate that α7nAChR is required for GTS‐21‐mediated protection and suggest that activation of the MEF2/PGC‐1α/HO‐1 axis underlies its regulatory effects on mitochondrial function. The apoptosis data shown in Figures 1 and 2 were derived from independent experiments. Therefore, minor variations in the absolute percentages of apoptotic cells are expected and reflect normal biological variability. Comparisons should be made only within the same experimental batch.

### 3.3 GTS‐21 upregulates PGC‐1α and HO‐1 via MEF2

**FIGURE 4:**
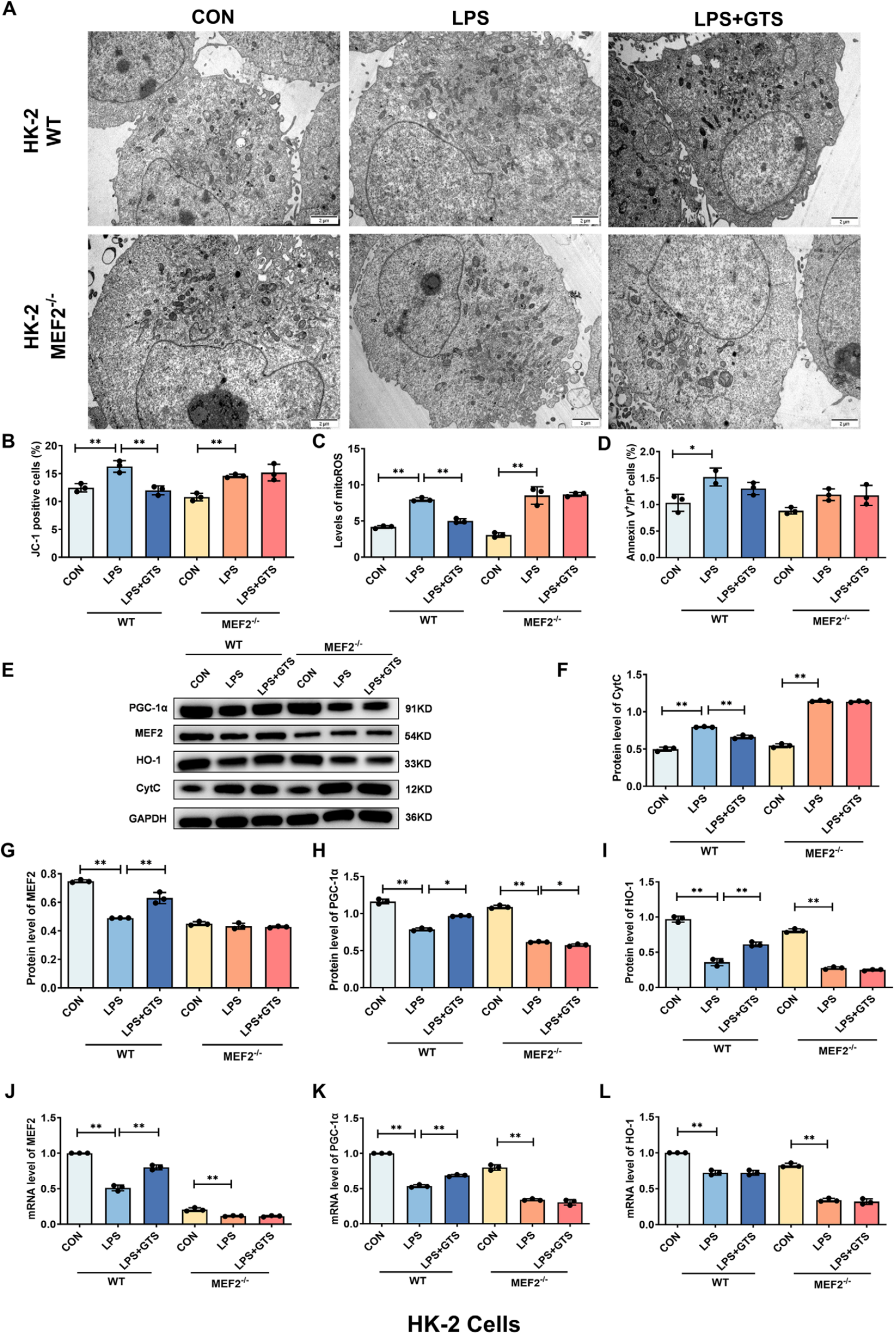
MEF2 contributes to GTS‐21‐mediated upregulation of PGC‐1α and HO‐1 in HK‐2 cells. (A) Representative TEM images of HK‐2 cells. Scale bar = 2 µm. (B–D) Effects of MEF2 deficiency on MMP, mito‐ROS levels and apoptosis in HK‐2 cells after LPS stimulation. (E–I) Representative Western blot images and quantitative analysis of Cyt c, MEF2, HO‐1 and PGC‐1α in MEF2‐deficient HK‐2 cells following LPS stimulation. (J–L) mRNA expression levels of MEF2, HO‐1 and PGC‐1α in MEF2‐deficient HK‐2 cells following LPS stimulation. Data are presented as mean ± SD. ^*^ *p* < .05, ^**^ *p* < .01.

**FIGURE 5:**
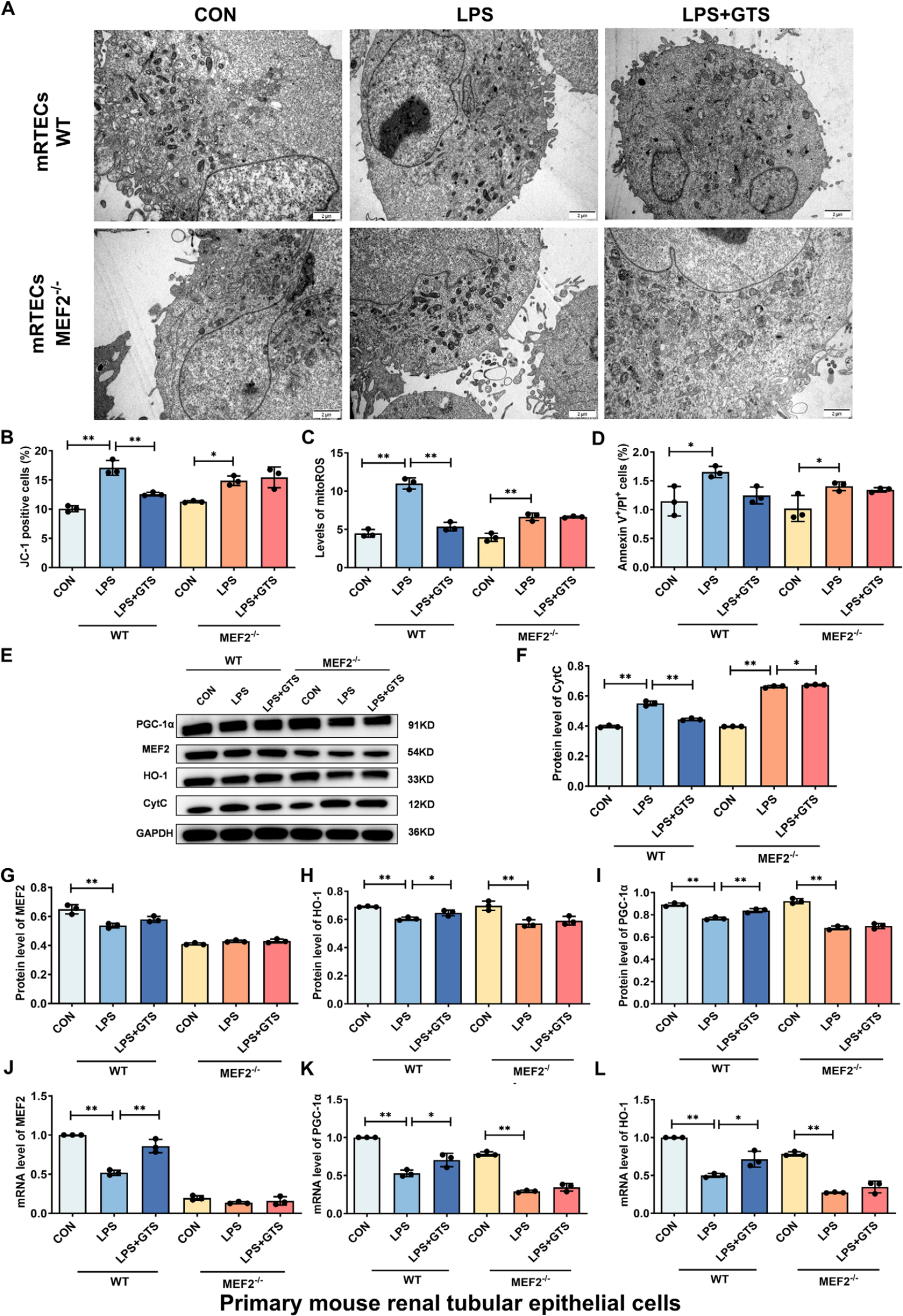
MEF2 contributes to GTS‐21‐mediated upregulation of PGC‐1α and HO‐1 in mRTECs. (A) Representative TEM images of mRTECs. Scale bar = 2 µm. (B–D) Effects of MEF2 deficiency on MMP, mito‐ROS levels and apoptosis in mRTECs following LPS stimulation. (E–I) Representative Western blot images and quantitative analysis of Cyt c, MEF2, HO‐1 and PGC‐1α in MEF2‐deficient mRTECs following LPS stimulation. (J–L) mRNA expression levels of MEF2, HO‐1 and PGC‐1α in MEF2‐deficient mRTECs following LPS stimulation. Data are presented as mean ± SD. ^*^ *p* < .05, ^**^ *p* < .01.

To investigate the downstream mechanisms of α7nAChR activation, the regulatory relationships among MEF2, PGC‐1α and HO‐1 were examined. As shown in Figures 2 and 3, GTS‐21 treatment significantly increased the expression of MEF2, PGC‐1α and HO‐1. To determine whether MEF2 is required for these effects, MEF2 knockdown was performed in HK‐2 cells and mRTECs using shRNA. Suppression of MEF2 expression was confirmed following sh‐MEF2 transduction. Notably, MEF2 knockdown markedly attenuated the protective effects of GTS‐21, as evidenced by impaired mitochondrial structural integrity, increased mitochondrial ROS levels, enhanced Cyt c release, and elevated apoptosis (Figures 4A–F and 5A–F). Furthermore, MEF2 deficiency significantly reduced the ability of GTS‐21 to upregulate PGC‐1α and HO‐1 expression (Figures 4E,G–L and 5E,G–L), indicating that MEF2 is an important mediator involved in GTS‐21‐mediated activation of these downstream targets. In addition, dual‐luciferase reporter assays demonstrated that MEF2 directly regulates PGC‐1α transcription, as MEF2 overexpression significantly enhanced PGC‐1α promoter activity (Figure S4). Collectively, these results suggest MEF2 plays a key role in linking α7nAChR activation to the regulation of PGC‐1α and HO‐1, thereby contributing to mitochondrial protection.

### 3.4 GTS‐21 ameliorates mitochondrial damage and promotes mitochondrial biogenesis in CLP‐induced mice

**FIGURE 6:**
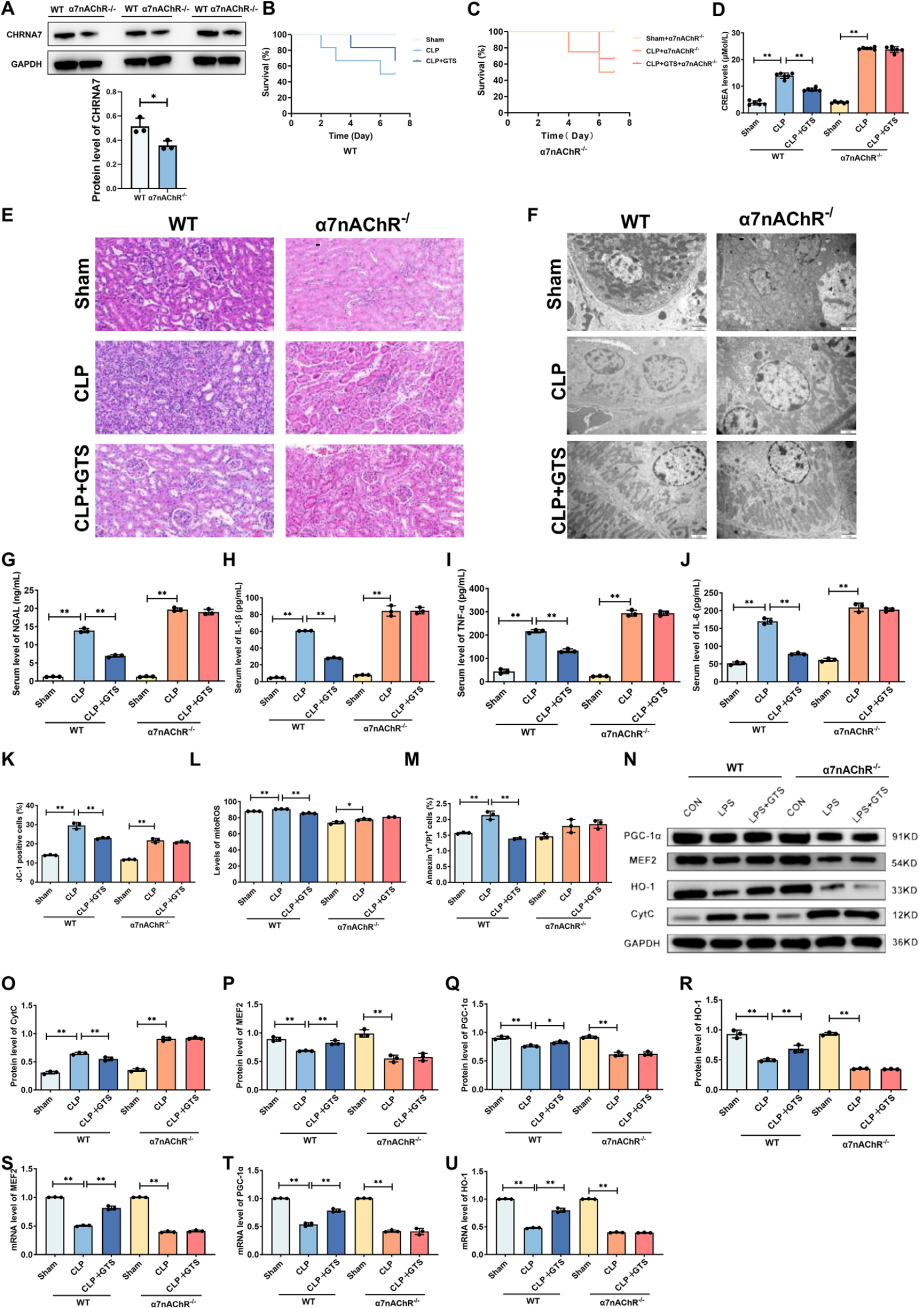
GTS‐21 ameliorates mitochondrial damage and promotes mitochondrial biogenesis in CLP‐induced mice. (A)Validation of α7nAChR deficiency in mouse kidney tissues. (B and C) Effects of GTS‐21 on survival in CLP‐induced mice. (*n* = 6) (D) Effects of α7nAChR on serum creatinine levels in CLP‐induced mice. (E) Representative H&E‐stained images of kidney tissues from CLP‐induced mice. Scale bar = 50 µm. (F) Representative TEM images of mitochondrial ultrastructure in kidney tissues from different experimental groups, including Sham, CLP and CLP + GTS‐21 mice. Scale bar = 2 µm. (G‐J) Effects of α7nAChR on serum levels of NGAL, IL‐1β, TNF‐α and IL‐6 in CLP‐induced mice. (K–M) Effects of α7nAChR on MMP, mito‐ROS levels and apoptosis in CLP‐induced mice. (N–R) Representative Western blot images and quantitative analysis of CytC, MEF2, HO‐1, and PGC‐1α in CLP‐induced mice. (T‐U) mRNA expression levels of MEF2, HO‐1 and PGC‐1α in CLP‐induced mice. Data are presented as mean ± SD. ^*^ *p* < .05, ^**^ *p* < .01. (*n* = 3mice per group).

To evaluate the protective effects of GTS‐21 in vivo, a CLP‐induced S‐AKI model was established, followed by GTS‐21 administration. Successful deletion of α7nAChR in mouse kidney tissues was confirmed by Western blot analysis, which demonstrated a significant reduction in α7nAChR protein expression in α7nAChR‐deficient mice compared with WT mice (Figure 6A). Compared with the sham group, CLP markedly reduced survival rates and increased serum creatinine levels, whereas GTS‐21 treatment significantly improved survival and decreased creatinine levels (Figure 6B–D). In contrast, α7nAChR‐deficient mice exhibited aggravated injury, as evidenced by higher mortality and creatinine levels, and the protective effects of GTS‐21 were abolished. Histopathological analysis showed that GTS‐21 significantly alleviated renal tissue injury in CLP mice (Figure 6E). TEM analysis further demonstrated that GTS‐21 preserved mitochondrial ultrastructure and increased cristae density following CLP (Figure 6F). Consistently, CLP induced a robust inflammatory response, as indicated by elevated serum levels of NGAL, IL‐1β, TNF‐α and IL‐6, whereas GTS‐21 significantly suppressed these increases (Figure 6G–J). In addition, GTS‐21 restored MMP (Figure 6K), reduced mitochondrial ROS levels (Figure 6L), and attenuated apoptosis (Figure 6 M–O). These protective effects were not observed in α7nAChR‐deficient mice. Consistent with the in vitro findings, GTS‐21 restored the expression of MEF2, PGC‐1α and HO‐1 in CLP‐treated wild‐type mice, whereas this effect was abolished in α7nAChR‐deficient mice (Figure 6 M,P–U).

Collectively, these results demonstrate that GTS‐21 confers renoprotection in CLP‐induced S‐AKI in an α7nAChR‐dependent manner, which is associated with activation of the MEF2/PGC‐1α/HO‐1 axis and subsequent improvement of mitochondrial function.

### 3.5 GTS‐21 ameliorates CLP‐induced injury in mice in a MEF2/PGC‐1α/HO‐1‐dependent manner

**FIGURE 7:**
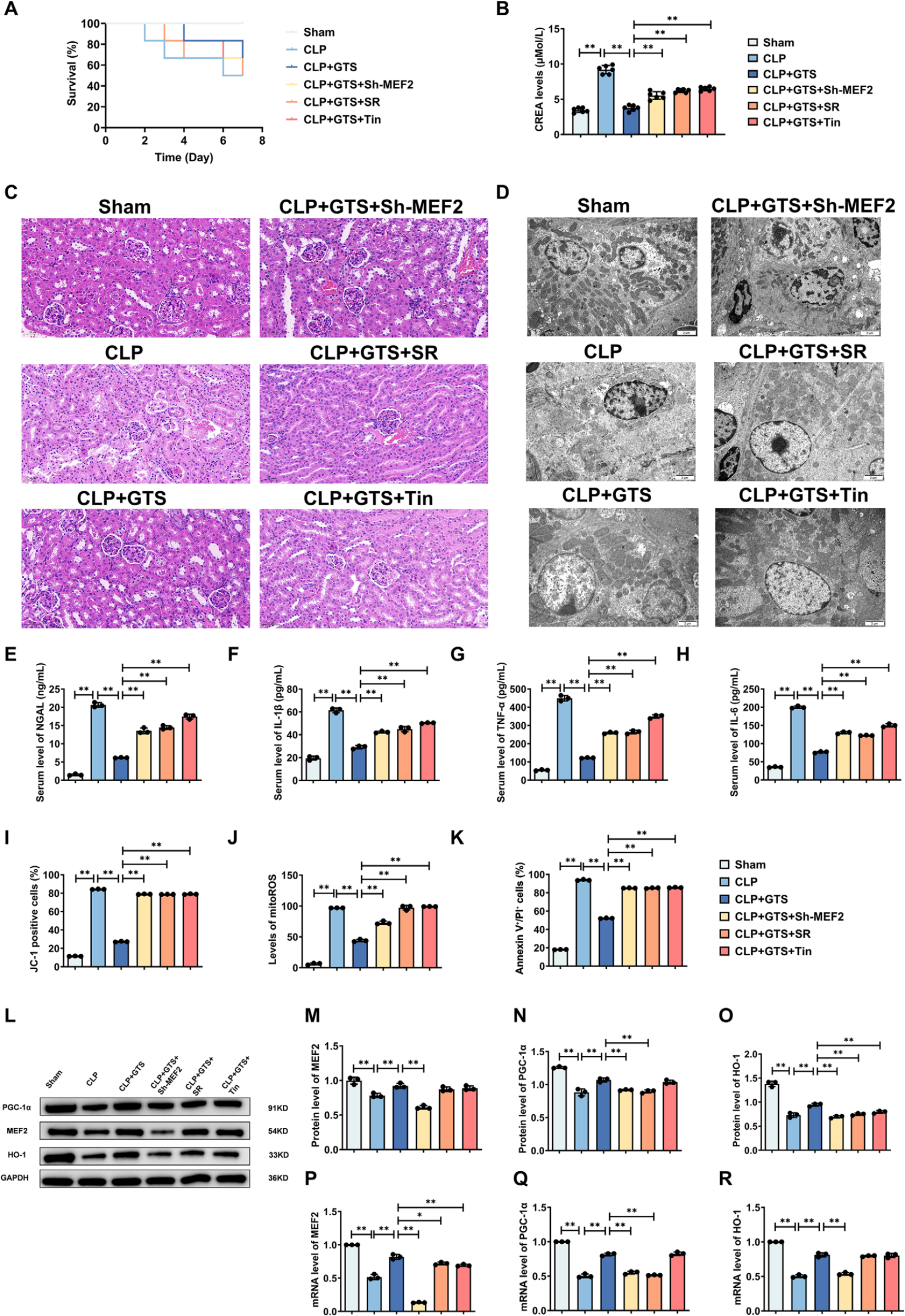
GTS‐21 ameliorates CLP‐induced injury in mice in a MEF2/PGC‐1α/HO‐1‐dependent manner. (A) Effects of MEF2 deficiency or inhibition of PGC‐1α and HO‐1 on survival in CLP‐induced mice. (B) Effects of MEF2 deficiency or inhibition of PGC‐1α and HO‐1 on serum creatinine levels in CLP‐induced mice. (*n* = 6) (C) Representative H&E‐stained images of kidney tissues from CLP‐induced mice. Scale bar = 50 µm. (D) Representative TEM images of kidney tissues from CLP‐induced mice. Scale bar = 2 µm. (E–H) Effects of MEF2 deficiency or inhibition of PGC‐1α and HO‐1 on serum levels of NGAL, IL‐1β, TNF‐α and IL‐6 in CLP‐induced mice. (I–K) Effects of MEF2 deficiency or inhibition of PGC‐1α and HO‐1 on MMP, mitochondrial ROS levels and apoptosis in CLP‐induced mice. (L–O) Representative Western blot images and quantitative analysis of MEF2, HO‐1 and PGC‐1α in CLP‐induced mice. (P–R) mRNA expression levels of MEF2, HO‐1 and PGC‐1α in CLP‐induced mice. Data are presented as mean ± SD. *p < .05, **p < .01. (*n* = 3mice per group).

To further validate the functional roles of MEF2, PGC‐1α and HO‐1 in vivo, MEF2 knockdown was performed in wild‐type mice, and pharmacological inhibition of PGC‐1α and HO‐1 was achieved using specific inhibitors. Disruption of these pathways markedly attenuated the protective effects of GTS‐21. Specifically, MEF2 knockdown or inhibition of PGC‐1α or HO‐1 significantly reduced GTS‐21‐mediated improvements in survival (Figure 7A), decreased serum creatinine levels (Figure 7B) and suppression of pro‐inflammatory cytokines (Figure 7E–H). In addition, the ability of GTS‐21 to restore MMP (Figure 7I), reduce mitochondrial ROS levels (Figure 7J) and inhibit apoptosis (Figure 7K) was significantly impaired. Histological and ultrastructural analyses further demonstrated that MEF2 knockdown or inhibition of PGC‐1α or HO‐1 abolished the protective effects of GTS‐21 on renal tissue injury and mitochondrial integrity in CLP‐induced mice (Figure 7C,D). Moreover, qPCR analysis showed that MEF2 knockdown eliminated GTS‐21‐induced upregulation of PGC‐1α and HO‐1 expression in kidney tissues (Figure 7L–R), indicating that MEF2 is required for the activation of these downstream targets. Collectively, these findings demonstrate that the renoprotective effects of GTS‐21 are dependent on the MEF2/PGC‐1α/HO‐1 axis, establishing this pathway as a critical mediator of α7nAChR signalling in S‐AKI.

## 4 DISCUSSION

In this study, we demonstrate that α7nAChR agonists mitigate mitochondrial dysfunction, suppress cellular apoptosis and attenuate renal inflammation by activating α7nAChR via a mechanism involving MEF2, HO‐1 and PGC‐1α, thereby preventing sepsis‐induced acute kidney injury (S‐AKI). S‐AKI remains a severe clinical condition with high morbidity and mortality, largely due to the lack of effective targeted therapies.^27^ Increasing evidence highlights the cholinergic anti‐inflammatory pathway as a critical regulator of systemic inflammation, in which α7nAChR activation suppresses pro‐inflammatory cytokine release and modulates immune responses.^28^ Emerging evidence indicates that α7nAChR activation confers cytoprotective effects in multiple pathologies by ameliorating mitochondrial dysfunction.^29^, ^30^ GTS‐21, a selective α7nAChR agonist, has demonstrated efficacy in attenuating inflammation, restoring mitochondrial homeostasis and ameliorating renal injury in experimental models of sepsis‐induced AKI.^28^, ^31^, ^32^ Consistent with these observations, our findings demonstrate that pharmacological activation of α7nAChR by GTS‐21 not only attenuates inflammation but also directly preserves mitochondrial integrity and function in both in vitro and in vivo models of S‐AKI. In vitro, lipopolysaccharide (LPS) was employed to induce cellular injury in HK‐2 cells and mRTECs. LPS treatment compromised outer mitochondrial membrane integrity, markedly diminished mitochondrial cristae density, elevated mitochondrial ROS levels and enhanced apoptotic activity. In vivo, caecal ligation and puncture (CLP) mice displayed decreased survival rates, aggravated renal histopathological damage, and concomitant mitochondrial dysfunction and inflammatory responses. Notably, GTS‐21 administration reversed these pathological alterations, whereas α7nAChR knockout abrogated its renoprotective effects in S‐AKI. Collectively, these findings indicate that GTS‐21 ameliorates mitochondrial injury, exerts anti‐apoptotic effects, and attenuates inflammation via an α7nAChR‐dependent mechanism in both cellular and animal models of sepsis‐induced AKI. Interestingly, SSR treatment significantly reduced Cyt c release but did not produce a comparable reduction in Annexin V‐positive cells. This apparent discrepancy may be attributable to the distinct biological processes reflected by these two indicators. Annexin V staining primarily detects phosphatidylserine externalization during early apoptosis, whereas Cyt c release represents mitochondrial outer membrane permeabilization and activation of the intrinsic apoptotic pathway. Therefore, SSR may preferentially preserve mitochondrial integrity and inhibit mitochondrial‐dependent apoptotic signalling without completely preventing early apoptotic membrane alterations induced by LPS. This observation suggests that the protective effects of SSR are more closely associated with modulation of mitochondrial apoptotic pathways. This finding further supports the notion that α7nAChR activation stabilizes mitochondrial homeostasis by specifically targeting the intrinsic apoptotic pathway.

However, the mechanisms by which α7nAChR activation ameliorates mitochondrial dysfunction in S‐AKI remain incompletely defined. Our findings further demonstrate that the renoprotective effects of α7nAChR activation are mediated through a coordinated transcriptional network involving MEF2, PGC‐1α, and HO‐1. Specifically, α7nAChR activation induces MEF2 upregulation, which subsequently drives the expression of PGC‐1α and HO‐1, thereby promoting mitochondrial biogenesis and reinforcing antioxidant defences. These results extend current understanding by identifying a previously underappreciated α7nAChR–MEF2/PGC‐1α/HO‐1 signalling axis in renal protection.

PGC‐1α is a central transcriptional coactivator that regulates mitochondrial biogenesis and oxidative metabolism through interactions with multiple transcription factors, including MEF2.^20^, ^22^ In the present study, activation of α7nAChR significantly increased both MEF2 and PGC‐1α expression, leading to improved mitochondrial function. Importantly, MEF2 knockdown completely abolished the protective effects of GTS‐21 in both HK‐2 cells and mRTECs, as well as in CLP‐induced mice, as evidenced by persistent mitochondrial damage, sustained inflammatory responses, and increased apoptosis. Similarly, pharmacological inhibition of PGC‐1α abrogated the renoprotective effects of α7nAChR activation. Mechanistically, dual‐luciferase reporter assays confirmed that MEF2 directly transactivates the PGC‐1α promoter, establishing a hierarchical regulatory relationship. Together, these findings identify the MEF2/PGC‐1α axis as a critical mediator of mitochondrial protection downstream of α7nAChR.

HO‐1, a stress‐inducible enzyme with potent antioxidant and anti‐inflammatory properties, has been widely implicated in the pathogenesis of S‐AKI.^33^ Substantial evidence indicates that HO‐1 ameliorates S‐AKI pathogenesis through multifaceted mechanisms, including suppression of apoptosis, oxidative stress, inflammatory responses and ferroptosis.^34^, ^35^, ^36^ α7nAChR activation has been shown to upregulate HO‐1 expression, thereby protecting against ischemia/reperfusion‐induced proximal tubular apoptosis.^37^ Furthermore, α7nAChR activation confers neuroprotection in cerebral ischemia through HO‐1‐mediated attenuation of neuroinflammation and oxidative stress.^38^ Consistent with previous reports, our results demonstrate that α7nAChR activation upregulates HO‐1 expression, thereby attenuating mitochondrial dysfunction, inflammation, and apoptosis. Notably, the protective effects of α7nAChR activation were abolished by either genetic deletion of α7nAChR or pharmacological inhibition of HO‐1, indicating that HO‐1 is functionally indispensable in this process. Furthermore, MEF2 knockdown prevented the upregulation of HO‐1, suggesting that HO‐1 operates downstream of MEF2 within this regulatory cascade. These findings position MEF2 as a central transcriptional hub coordinating both mitochondrial biogenesis and antioxidant defence programmes.

It is noteworthy that manufacturer‐reported potency rankings indicate that SSR180711 and AR‐R17779 exhibit higher intrinsic α7nAChR agonistic potency than GTS‐21 in receptor‐binding or electrophysiological assays. However, these values are typically obtained from heterologous expression systems using rapid ion channel or calcium flux measurements, which primarily reflect immediate receptor activation.

In contrast, our study evaluated functional cytoprotective outcomes in HK‐2 cells under LPS‐induced inflammatory stress, including attenuation of apoptosis and preservation of mitochondrial function over extended time periods. Differences in cellular context, receptor expression levels, downstream signalling coupling, and experimental endpoints may account for the observed divergence between intrinsic receptor potency and functional efficacy. Moreover, as a partial α7nAChR agonist, GTS‐21 may exert more stable anti‐inflammatory and cytoprotective effects under pathological conditions despite its relatively lower intrinsic potency. Therefore, the apparent discrepancy likely reflects differences in experimental systems and functional outcomes rather than inconsistencies in pharmacological activity.

Several limitations of this study should also be acknowledged. First, although GTS‐21 significantly attenuated renal injury, mitochondrial dysfunction, apoptosis and systemic inflammatory responses in CLP‐induced S‐AKI, the improvement in survival did not reach statistical significance. This may be attributed to the multifactorial nature of sepsis, in which survival outcomes are influenced not only by renal injury but also by systemic inflammation, cardiovascular dysfunction and multiple organ failure. In addition, the relatively small sample size used in the in vivo experiments, particularly for survival analysis, may have limited the statistical power to detect significant differences between groups. Furthermore, only a single dose and administration regimen of GTS‐21 was evaluated in the present study, which may not represent the optimal therapeutic strategy for improving long‐term survival outcomes. Future studies involving larger cohorts, dose‐escalation strategies, and repeated administration protocols are warranted to further validate the robustness of our findings and clarify the therapeutic window and translational potential of GTS‐21 in S‐AKI.

## 5 CONCLUSIONS

Collectively, our findings demonstrate that activation of α7nAChR confers robust protection against sepsis‐induced renal injury by preserving mitochondrial homeostasis, suppressing apoptosis and attenuating inflammatory responses. Mechanistically, these effects are mediated through a MEF2‐dependent transcriptional program that drives the activation of the PGC‐1α/HO‐1 axis, thereby coordinating mitochondrial biogenesis and antioxidant defence. These results identify the α7nAChR–MEF2/PGC‐1α/HO‐1 signalling pathway as a critical regulator of mitochondrial function in S‐AKI and highlight α7nAChR as a promising therapeutic target for the treatment of septic kidney injury.

## AUTHOR CONTRIBUTIONS

Yu‐Jia Tang, Hui‐Ying Liu, Na‐Qi Li, Xin Zhang, Kai Kang, Hong‐Liang Wang and Yang Gao conducted the literature search, conceived and designed the study, completed animal experiments, performed statistical analysis, analysed and discussed the results, and prepared, edited, and reviewed the manuscript. Yi‐Lu Lin, Yan Zhang, Yao Li, Jia‐Le Deng, Qing‐Min Meng, Yi‐Jin Tang, Zi‐Yue Zhang and Si‐Han Guan contributed to the literature search, completion of animal experiments, data acquisition and compilation, statistical analysis, analysis and discussion of the results and manuscript preparation. All authors read and approved the final article.

## CONFLICT OF INTEREST STATEMENT

The authors declare no conflicts of interest.

## ETHICS STATEMENT

The study protocol was reviewed and approved by the Ethics Committee of the Sixth Affiliated Hospital of Harbin Medical University (IRB number: LC2024‐058).

## Supporting information



Supporting Information

Supporting Information

## Data Availability

The authors are committed to providing the raw data that support the conclusion of this article without any reservation.
