# Supplementary material for: α7nAChR agonist GTS‐21 ameliorates sepsis‐induced acute kidney injury via MEF2/PGC‐1α/HO‐1 axis in mice
Source: Clin Transl Med. 2026 Jun 23;16(7):e70726. doi: 10.1002/ctm2.70726 (PMC13287963; doi:10.1002/ctm2.70726)
Supplement: Supplementary file 1 — Supporting Information [file CTM2-16-e70726-s001.pdf]

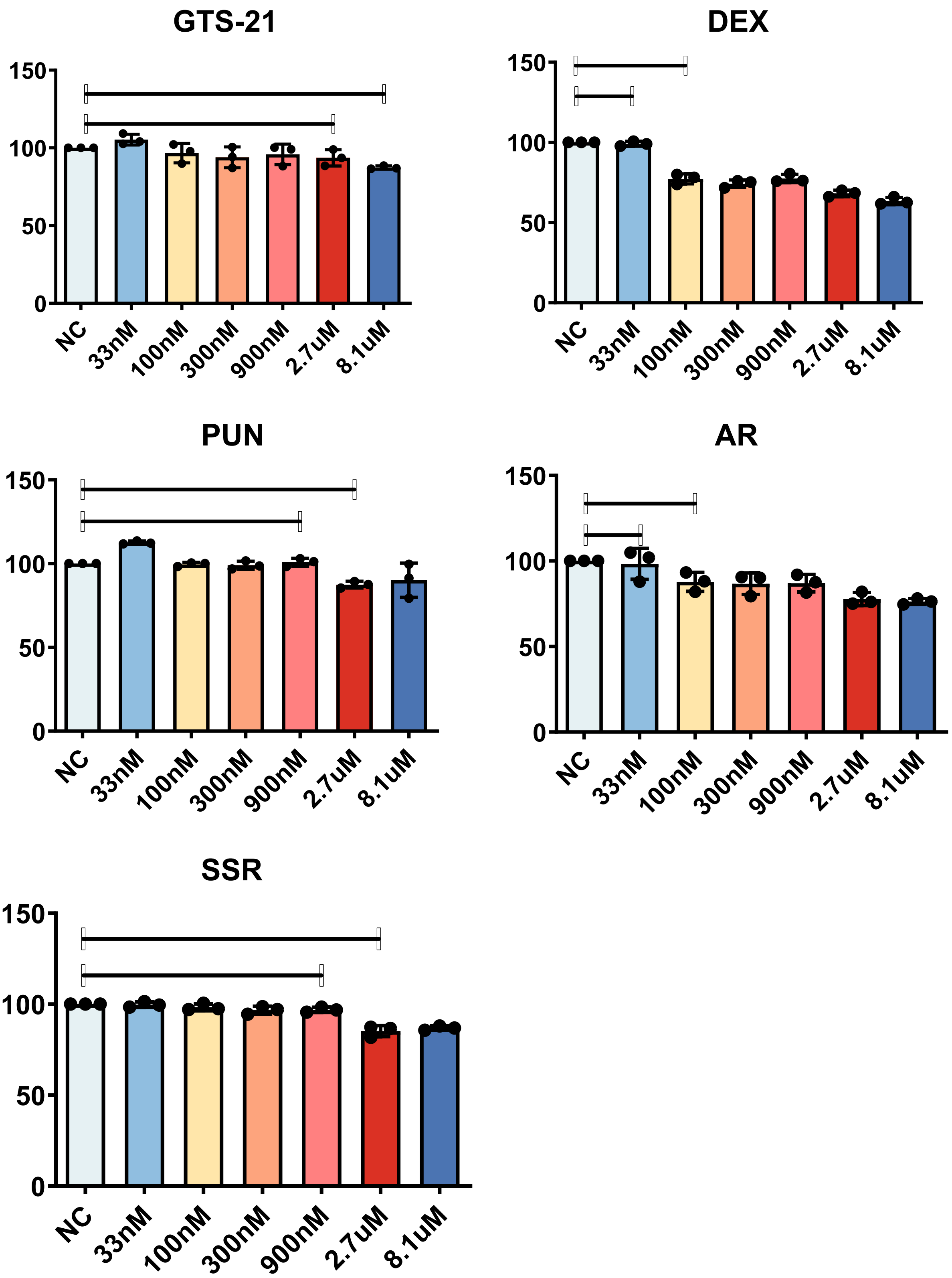

Supplementary Fig. 1. Determination of optimal drug concentrations using CCK-8 assays.

HK-2 cells were treated with different concentrations of α7nAChR agonists for 24 h, and cell viability was evaluated using CCK-8 assays. Concentrations used in subsequent experiments were selected based on the highest concentrations that did not significantly affect cell viability.

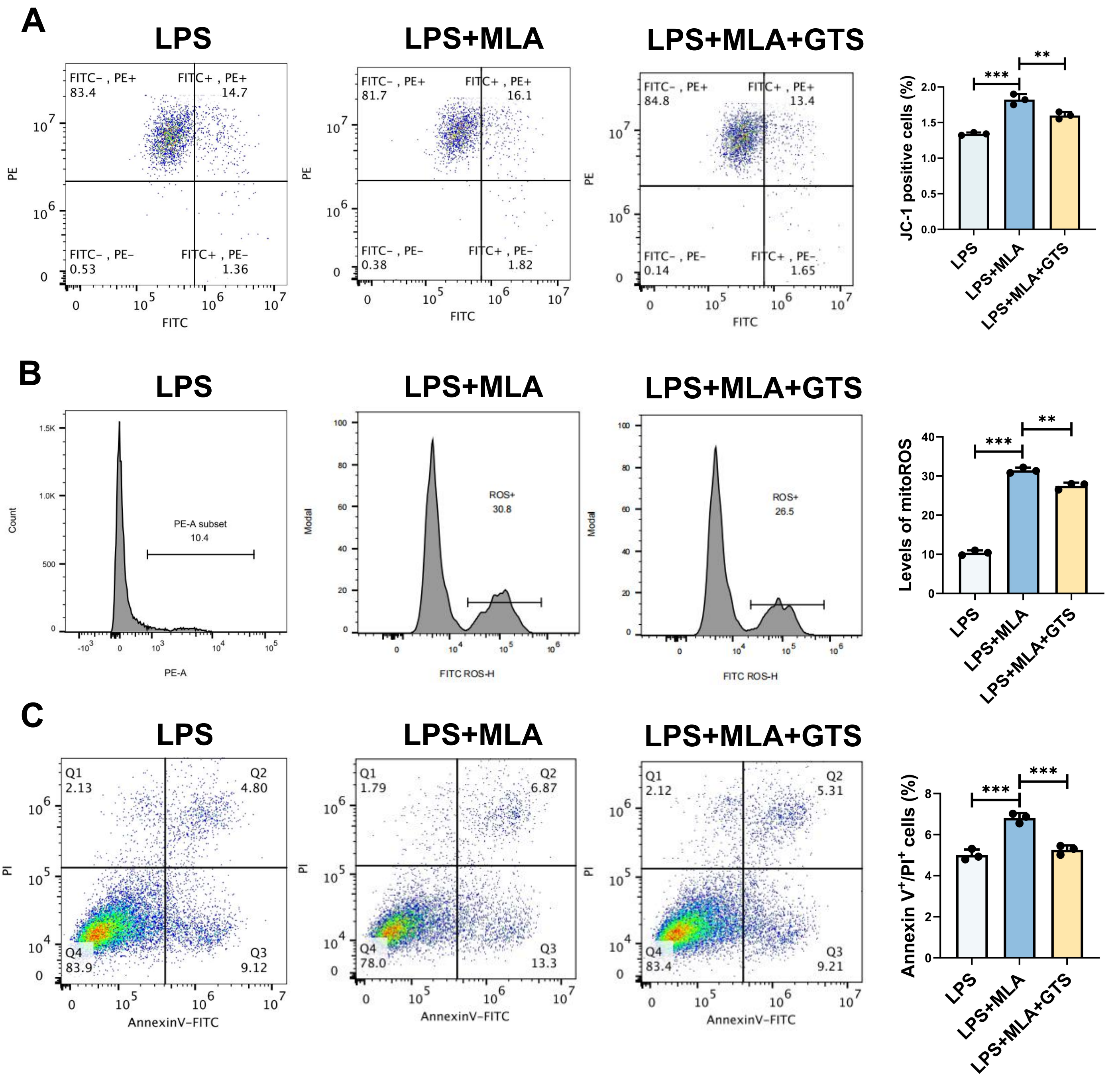

Supplementary Fig. 2. Effects of the selective α7nAChR antagonist methyllycaconitine (MLA) on mitochondrial function and apoptosis in HK-2 cells following LPS stimulation.

(A) Representative flow cytometry plots and quantitative analysis of mitochondrial membrane potential (JC-1) in HK-2 cells treated with LPS, LPS+MLA, and LPS+MLA+GTS-21.

(B) Representative flow cytometry histograms and quantitative analysis of mitochondrial ROS (mitoROS) levels in HK-2 cells under the indicated treatments.

(C) Representative flow cytometry plots and quantitative analysis of apoptosis in HK-2 cells following different treatments. Data are presented as mean ± SD. $^{**}P < 0.01$, $^{***}P < 0.001$.

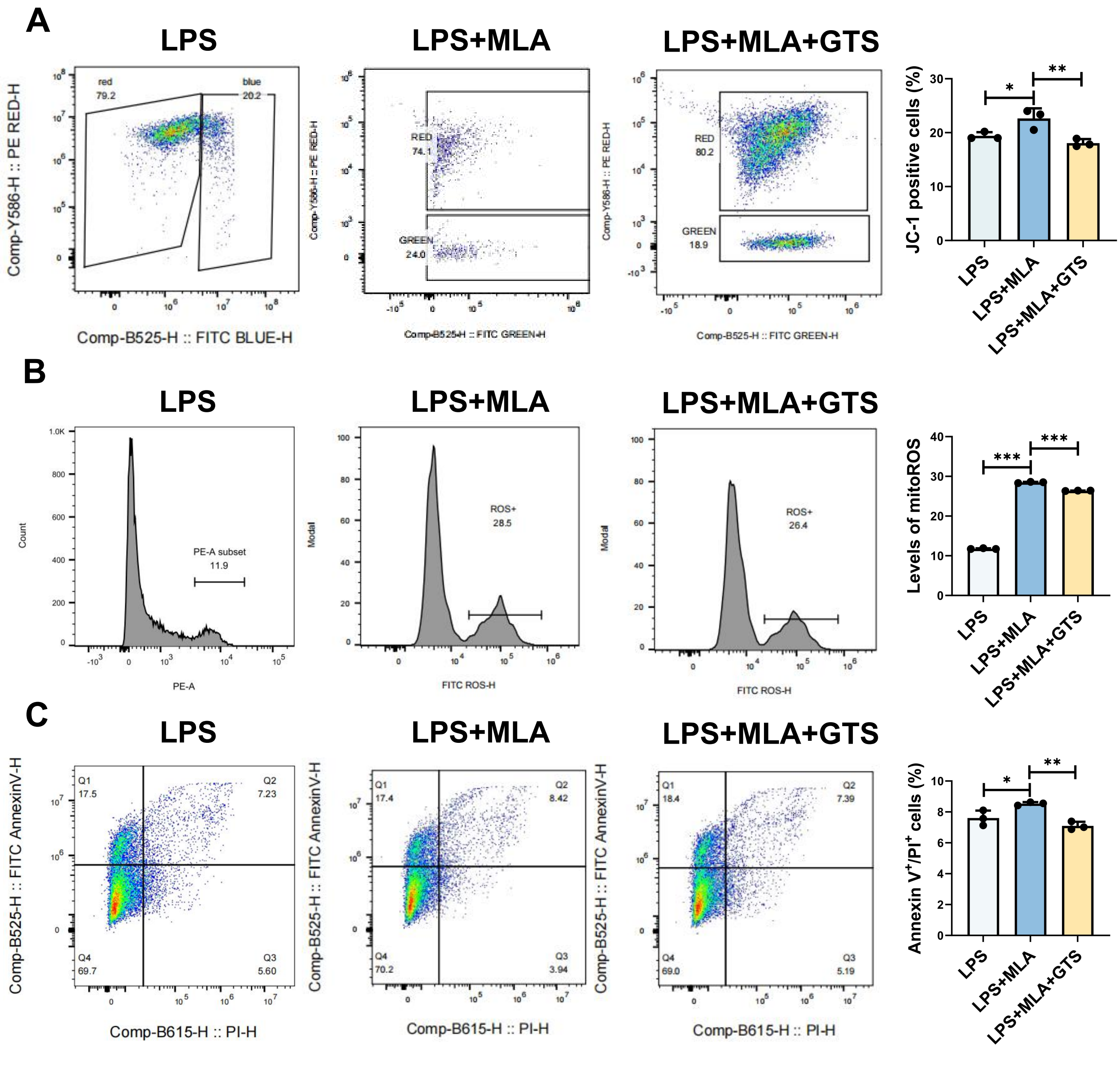

Supplementary Fig. 3. Effects of the selective α7nAChR antagonist methyllycaconitine (MLA) on mitochondrial function and apoptosis in mRTECs following LPS stimulation.

(A) Representative flow cytometry plots and quantitative analysis of mitochondrial membrane potential (JC-1) in mRTECs treated with LPS, LPS+MLA, and LPS+MLA+GTS-21.

(B) Representative flow cytometry histograms and quantitative analysis of mitochondrial ROS (mitoROS) levels in mRTECs under the indicated treatments.

(C) Representative flow cytometry plots and quantitative analysis of apoptosis in mRTECs following different treatments. Data are presented as mean ± SD. *P < 0.05, **P < 0.01, ***P < 0.001.

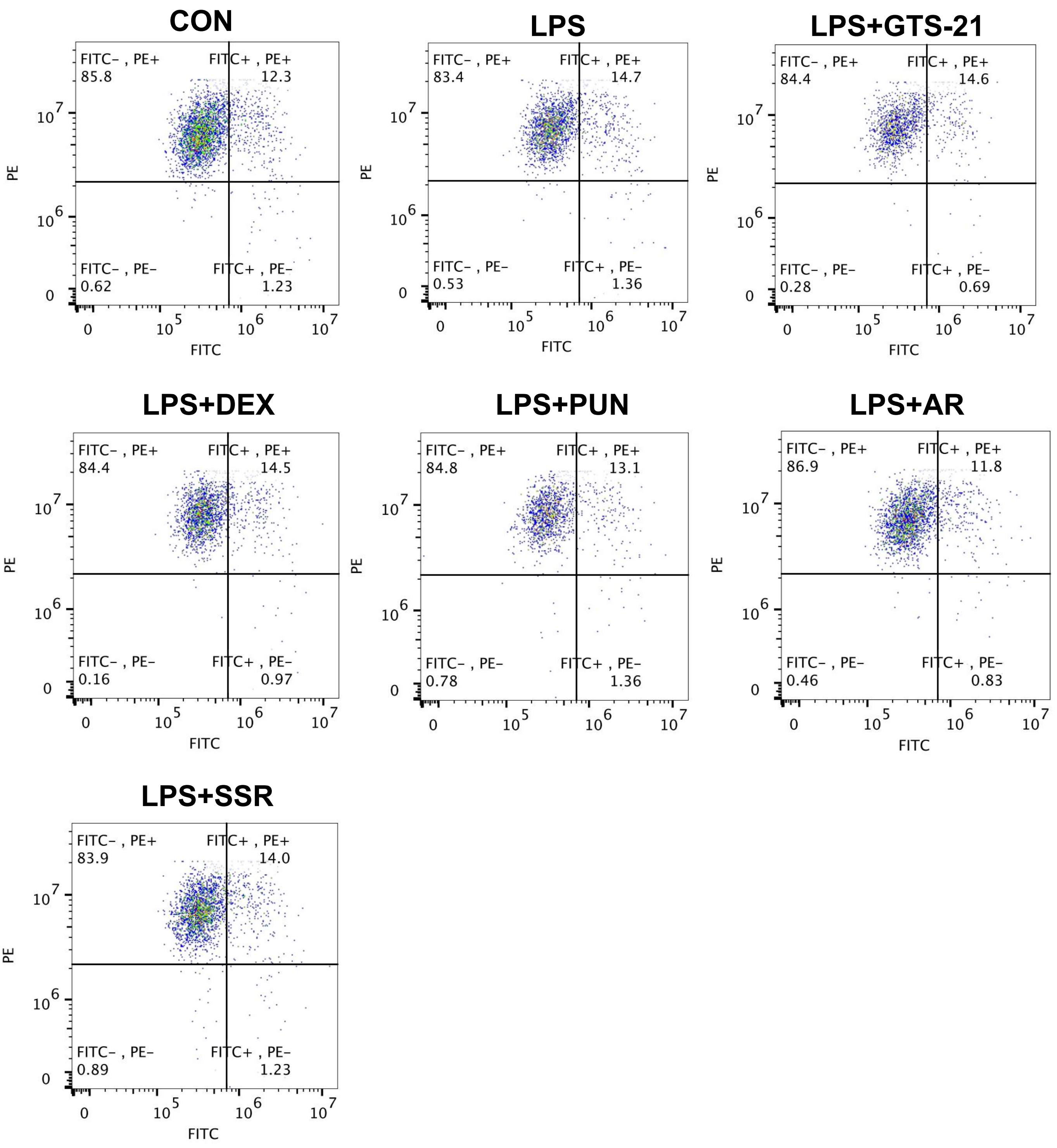

Fig.1. α7nAChR agonists attenuate LPS-induced renal tubular cytotoxicity in vitro.

(B) Effects of α7nAChR agonists on MMP in HK-2 cells.

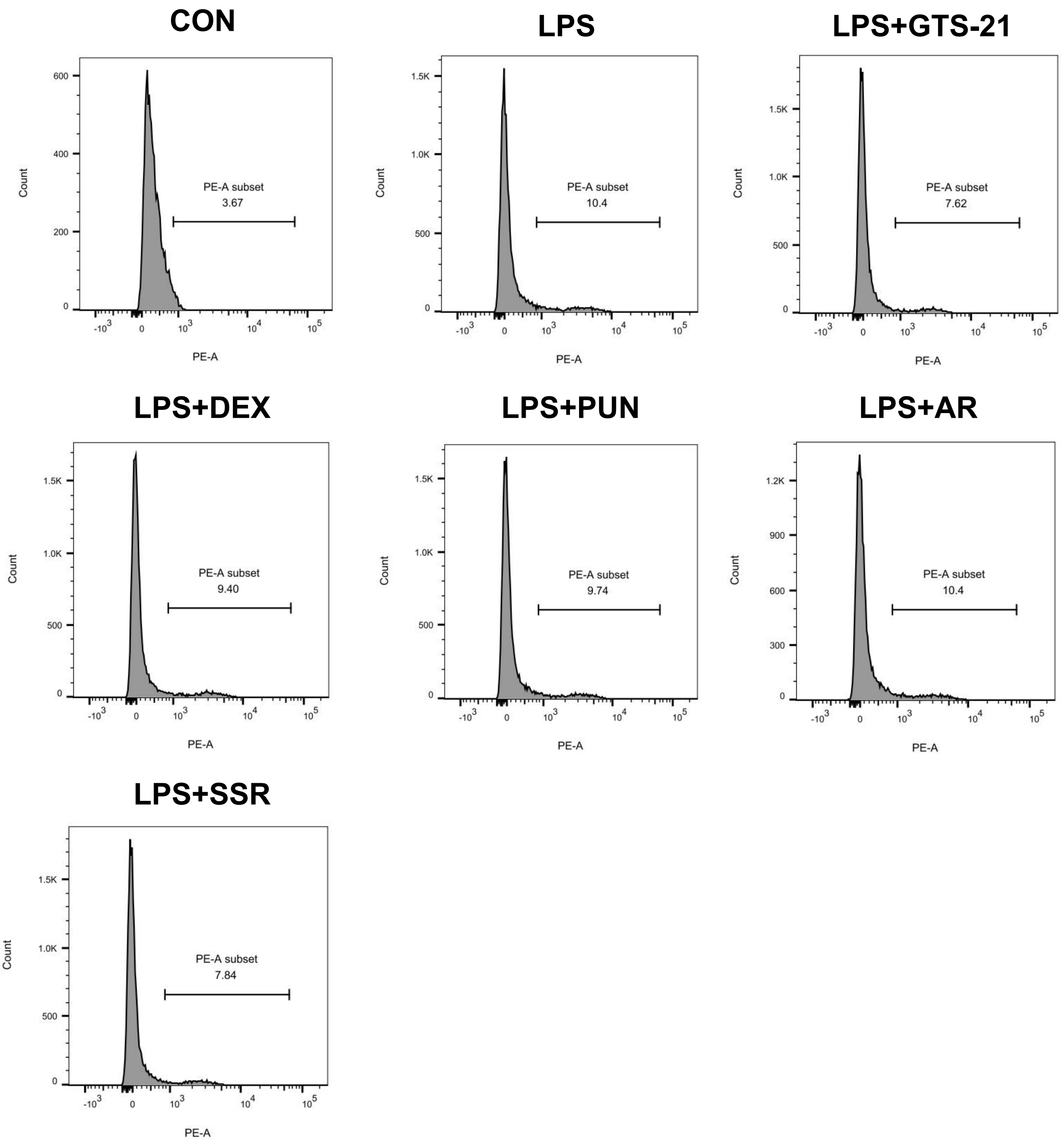

Fig.1. α7nAChR agonists attenuate LPS-induced renal tubular cytotoxicity *in vitro*.
(C) Effects of α7nAChR agonists on mito-ROS levels in HK-2 cells.

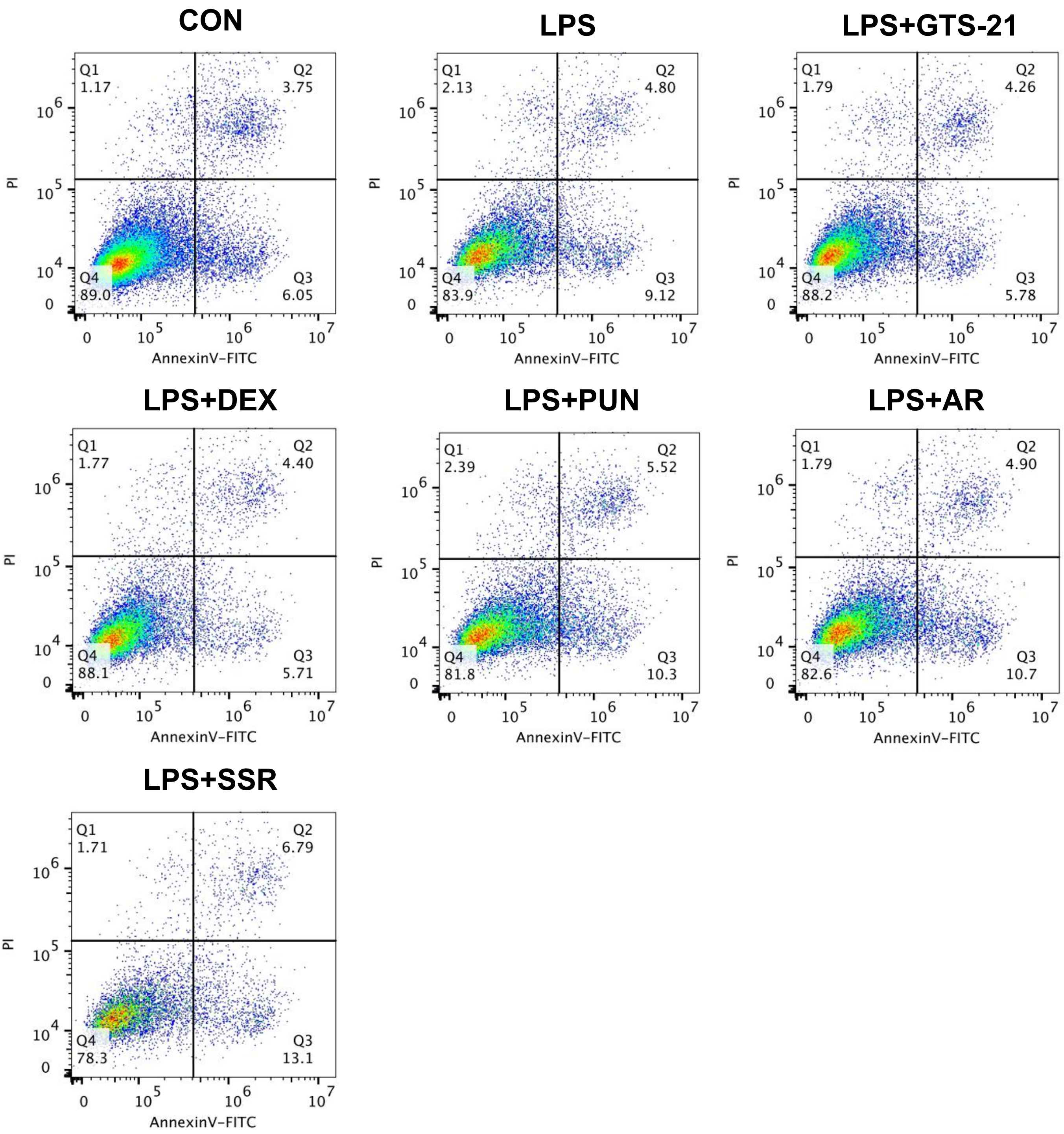

Fig.1. α7nAChR agonists attenuate LPS-induced renal tubular cytotoxicity *in vitro*.

(D) Effects of α7nAChR agonists on apoptosis in HK-2 cells.

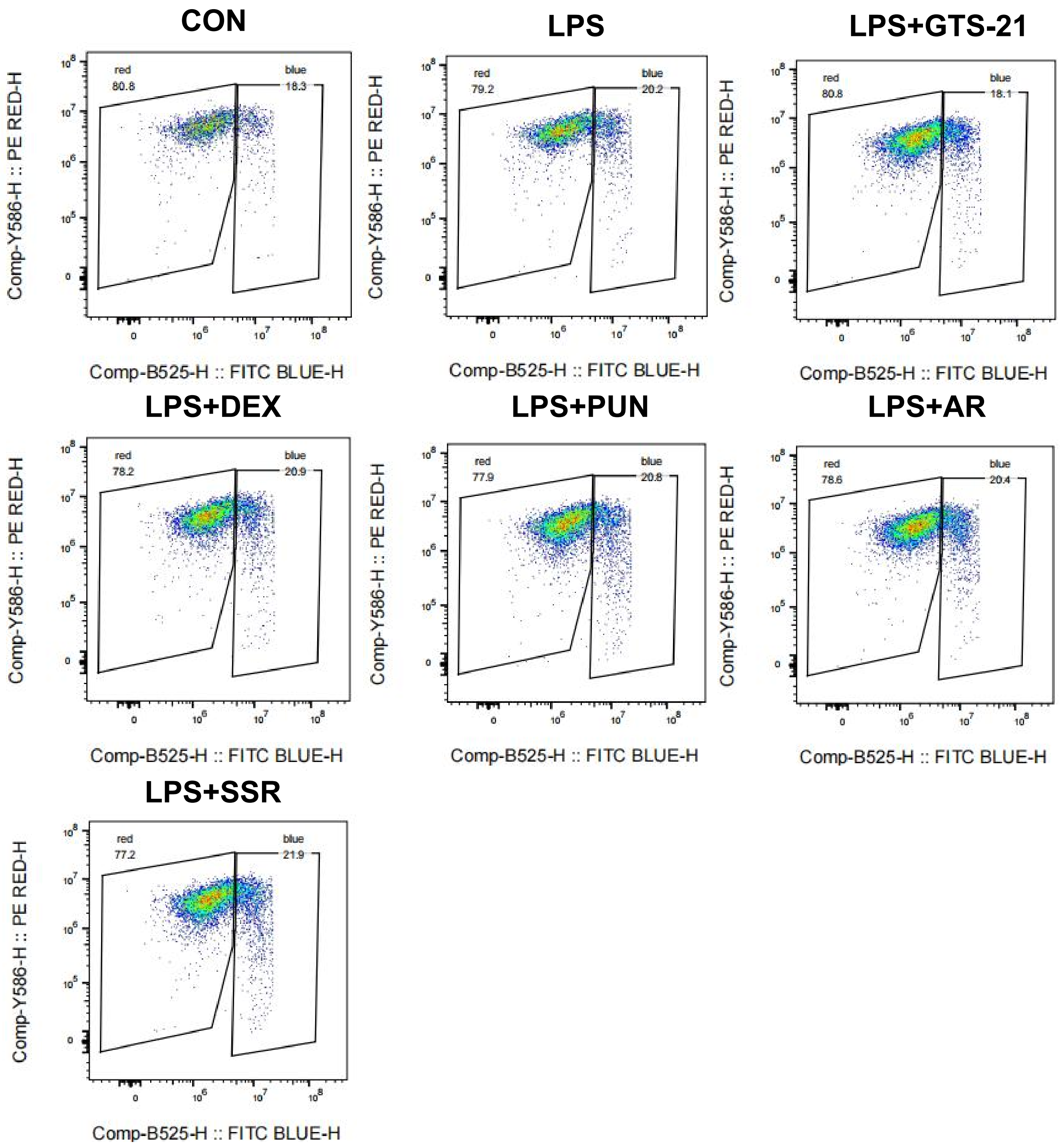

Fig.1. α7nAChR agonists attenuate LPS-induced renal tubular cytotoxicity *in vitro*.
(G) Effects of α7nAChR agonists on MMP in mRTECs.

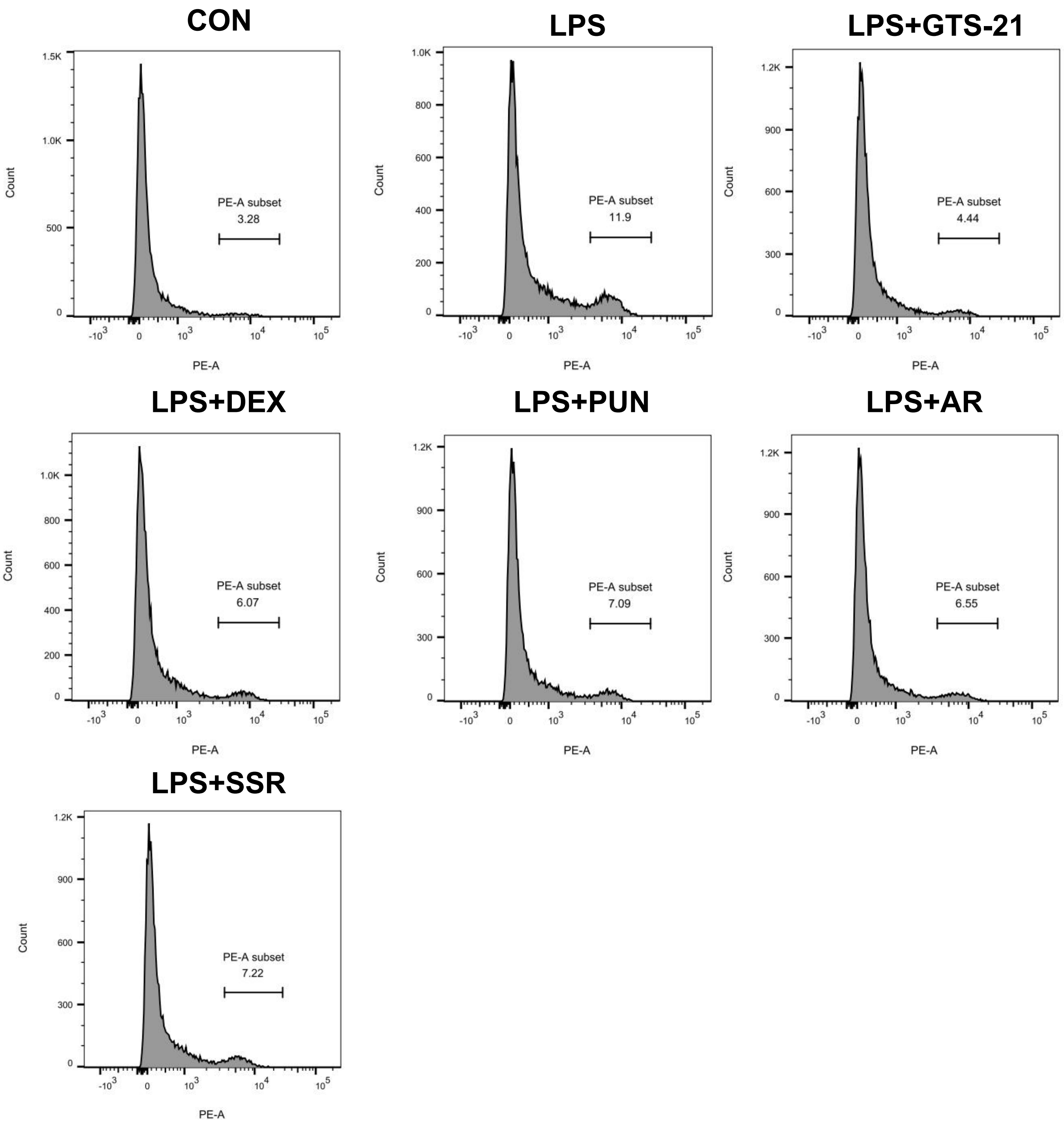

Fig.1. α7nAChR agonists attenuate LPS-induced renal tubular cytotoxicity *in vitro*.
(H) Effects of α7nAChR agonists on mito-ROS levels in mRTECs.

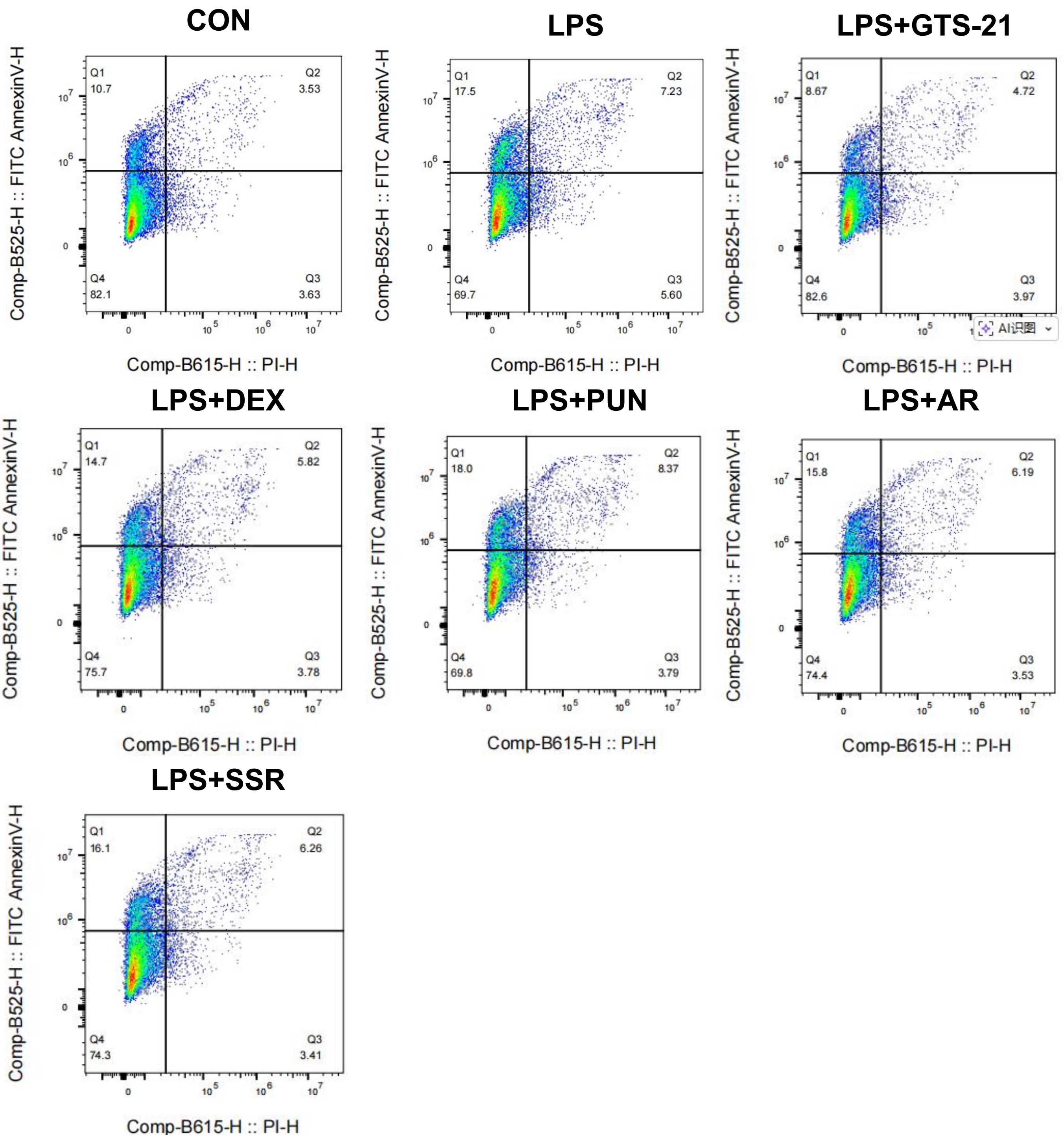

Fig.1. α7nAChR agonists attenuate LPS-induced renal tubular cytotoxicity *in vitro*.
(I) Effects of α7nAChR agonists on apoptosis in mRTECs.

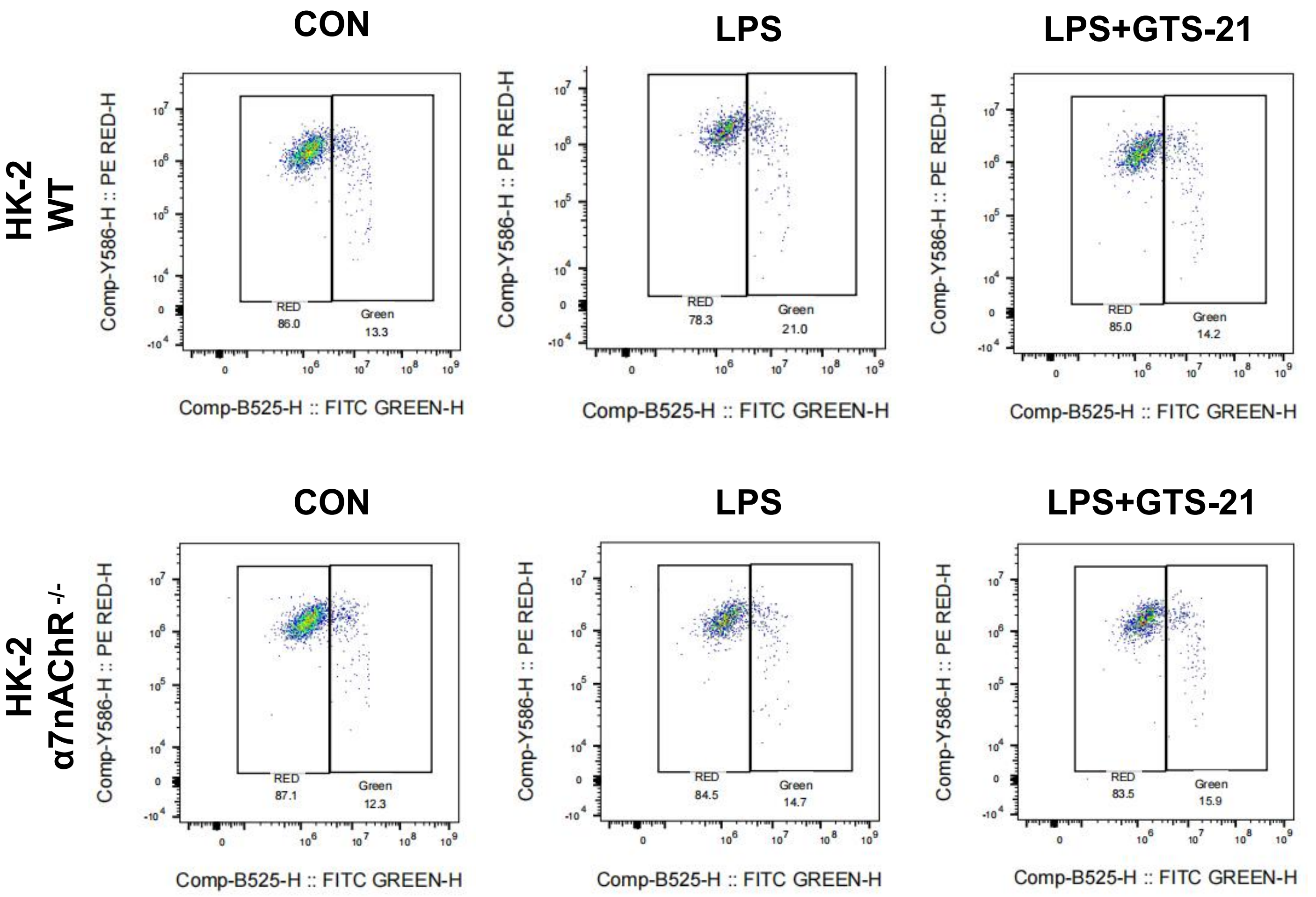

Fig.2. α7nAChR knockout reverses the protective effects of GTS-21 against LPS-induced injury in HK-2 cells.
(B) Effects of α7nAChR agonists on MMP in HK-2.

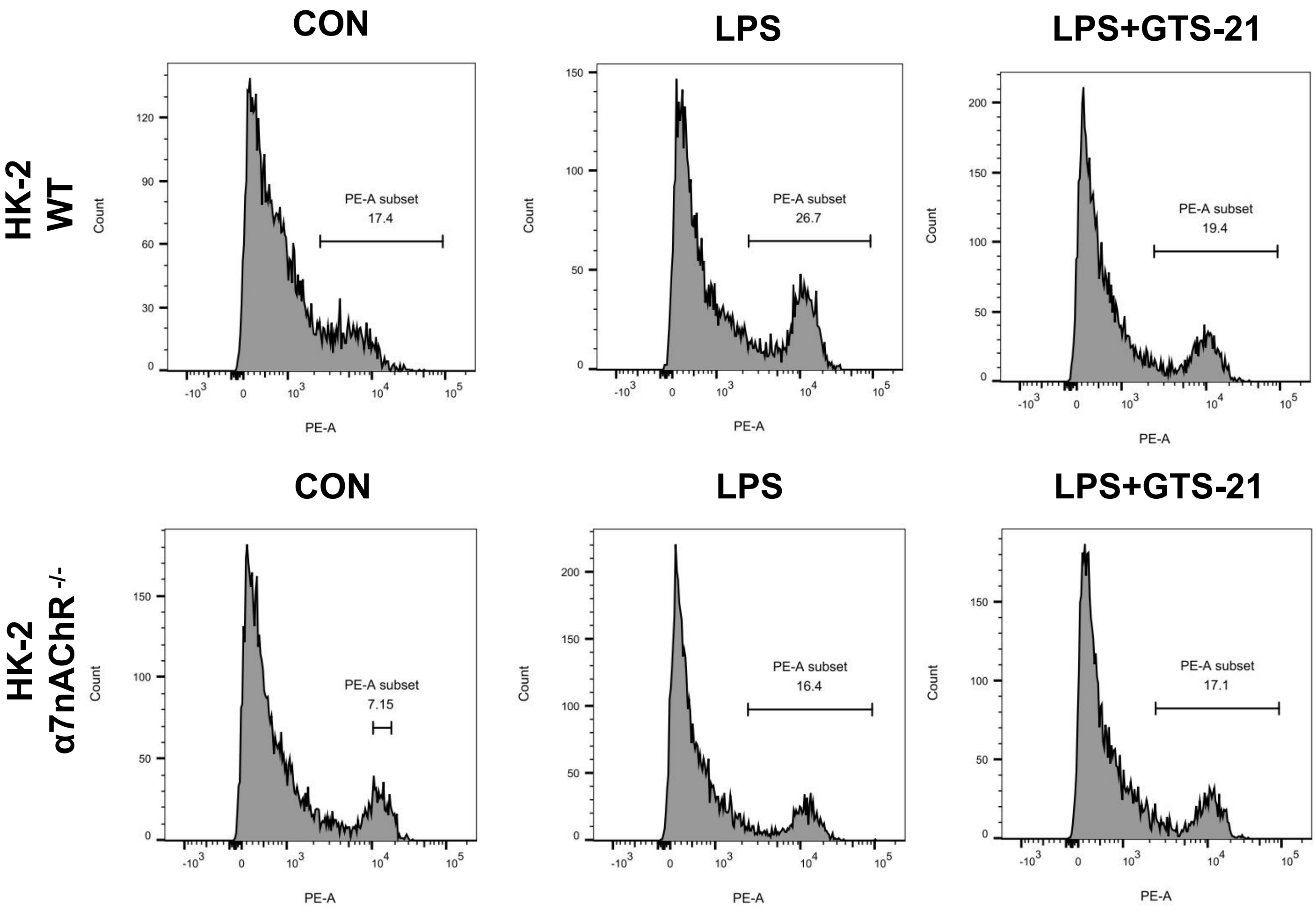

Fig.2. α7nAChR knockout reverses the protective effects of GTS-21 against LPS-induced injury in HK-2 cells.
(C) Effects of α7nAChR agonists on mito-ROS levels in HK-2.

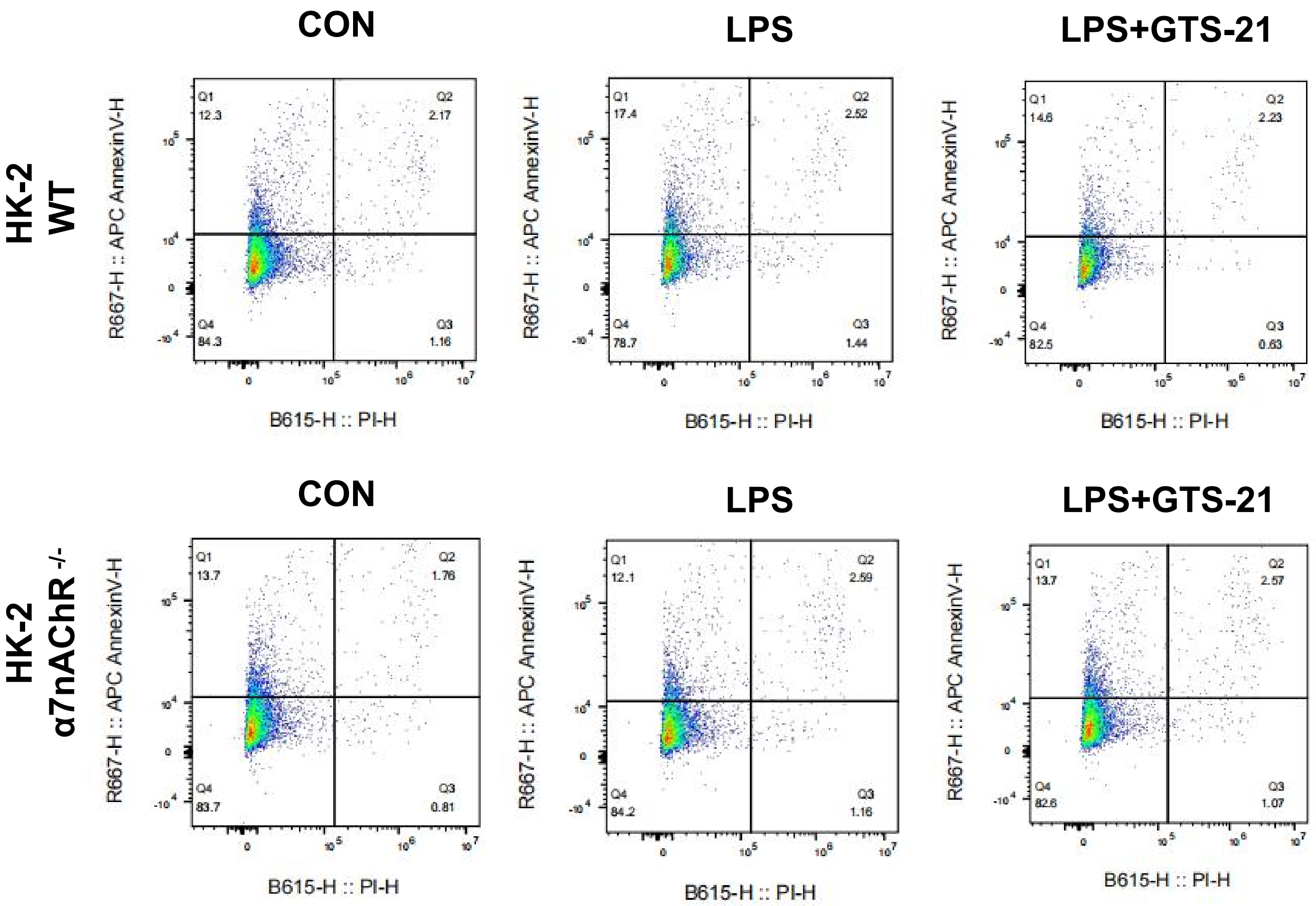

Fig.2. α7nAChR knockout reverses the protective effects of GTS-21 against LPS-induced injury in HK-2 cells.

(D) Effects of α7nAChR agonists on apoptosis in HK-2.

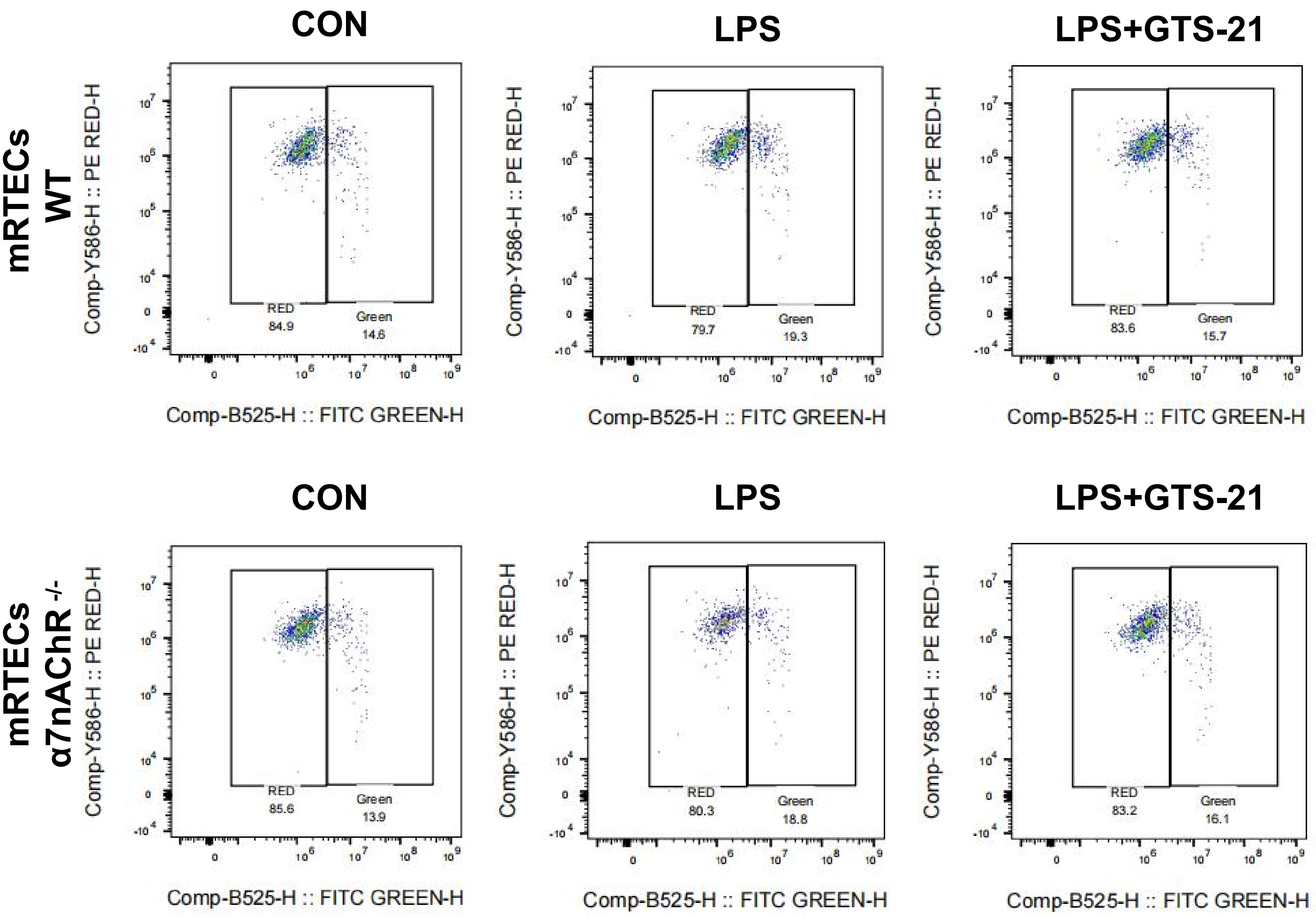

Fig.3. α7nAChR knockout reverses the protective effects of GTS-21 against LPS-induced injury in mRTECs.

(B) Effects of α7nAChR agonists on MMP in mRTECs.

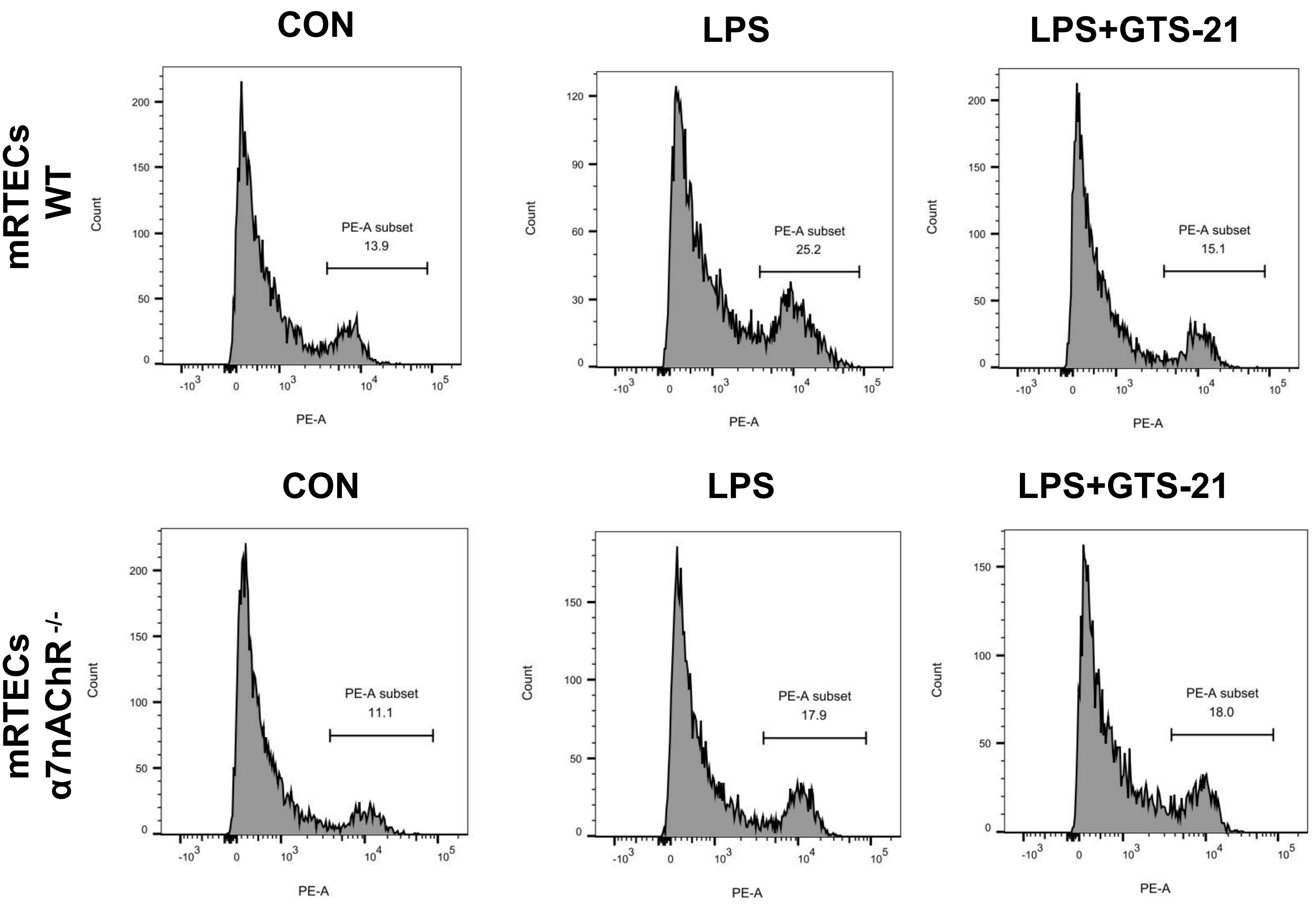

Fig.3. α7nAChR knockout reverses the protective effects of GTS-21 against LPS-induced injury in mRTECs.

(C) Effects of α7nAChR agonists on mito-ROS levels in mRTECs.

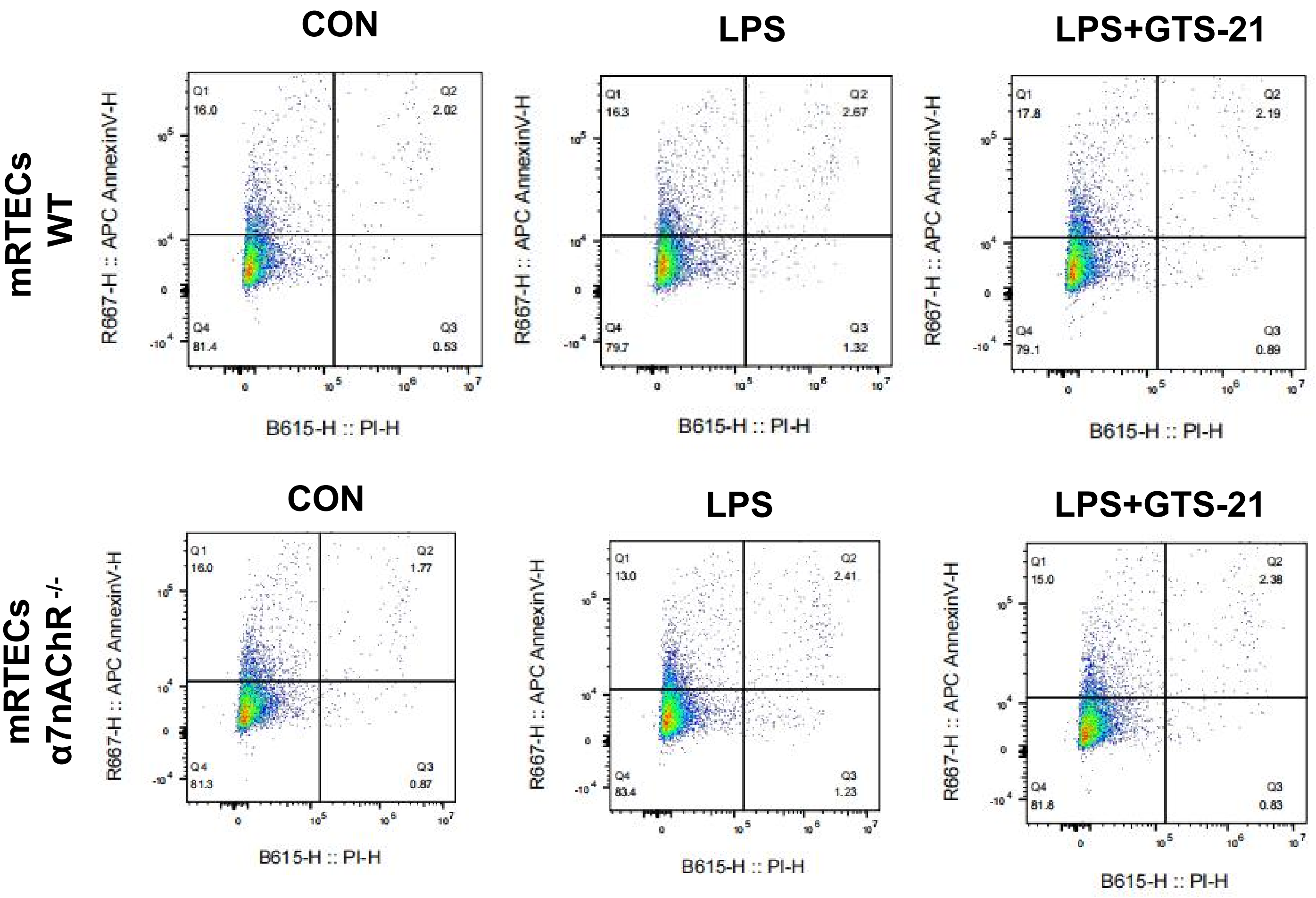

Fig.3. α7nAChR knockout reverses the protective effects of GTS-21 against LPS-induced injury in mRTECs.

(D) Effects of α7nAChR agonists on apoptosis in mRTECs.

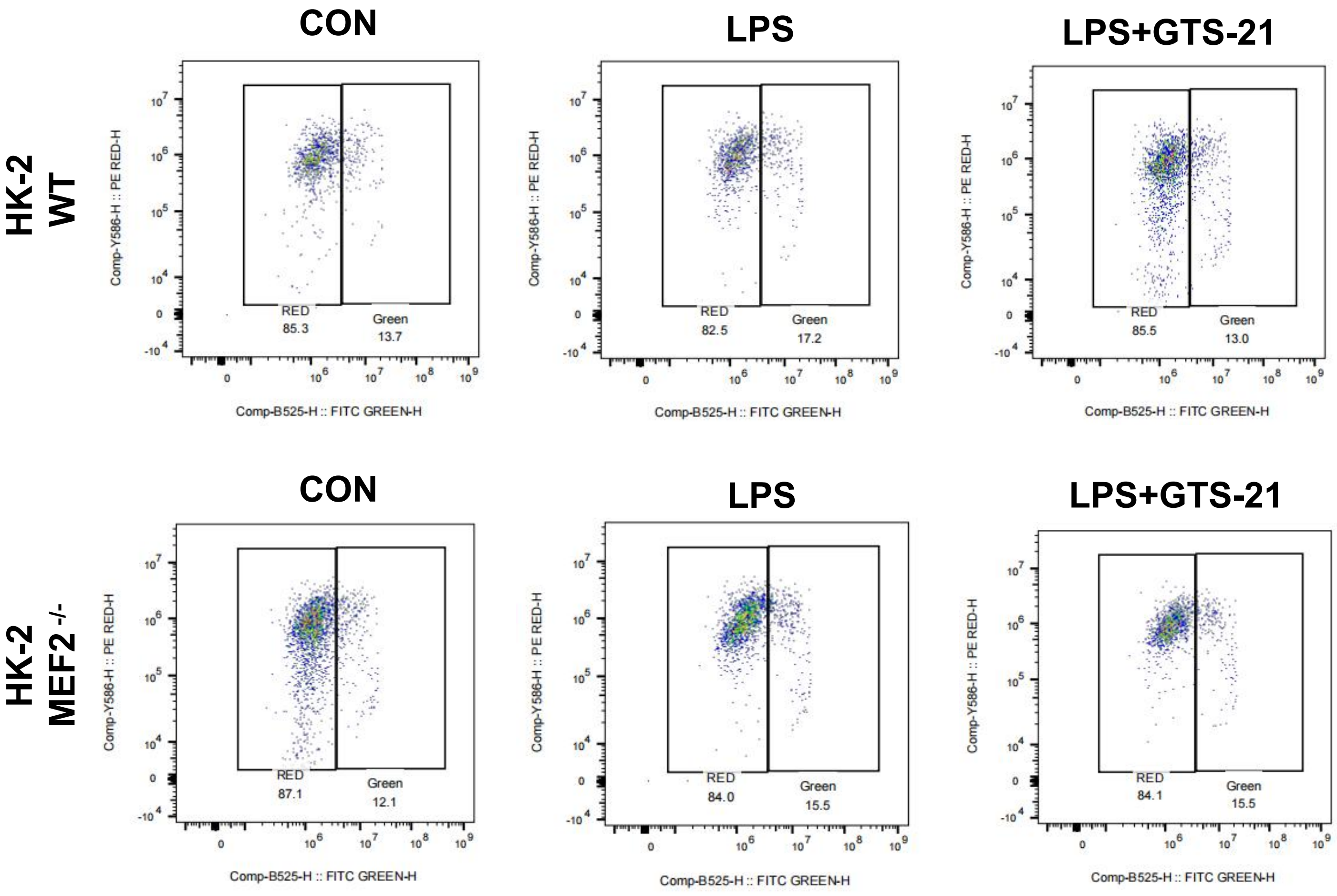

Fig.4. GTS-21 upregulates PGC-1α and HO-1 via MEF2 in HK-2 cells.
(B) Effects of α7nAChR agonists on MMP in HK-2.

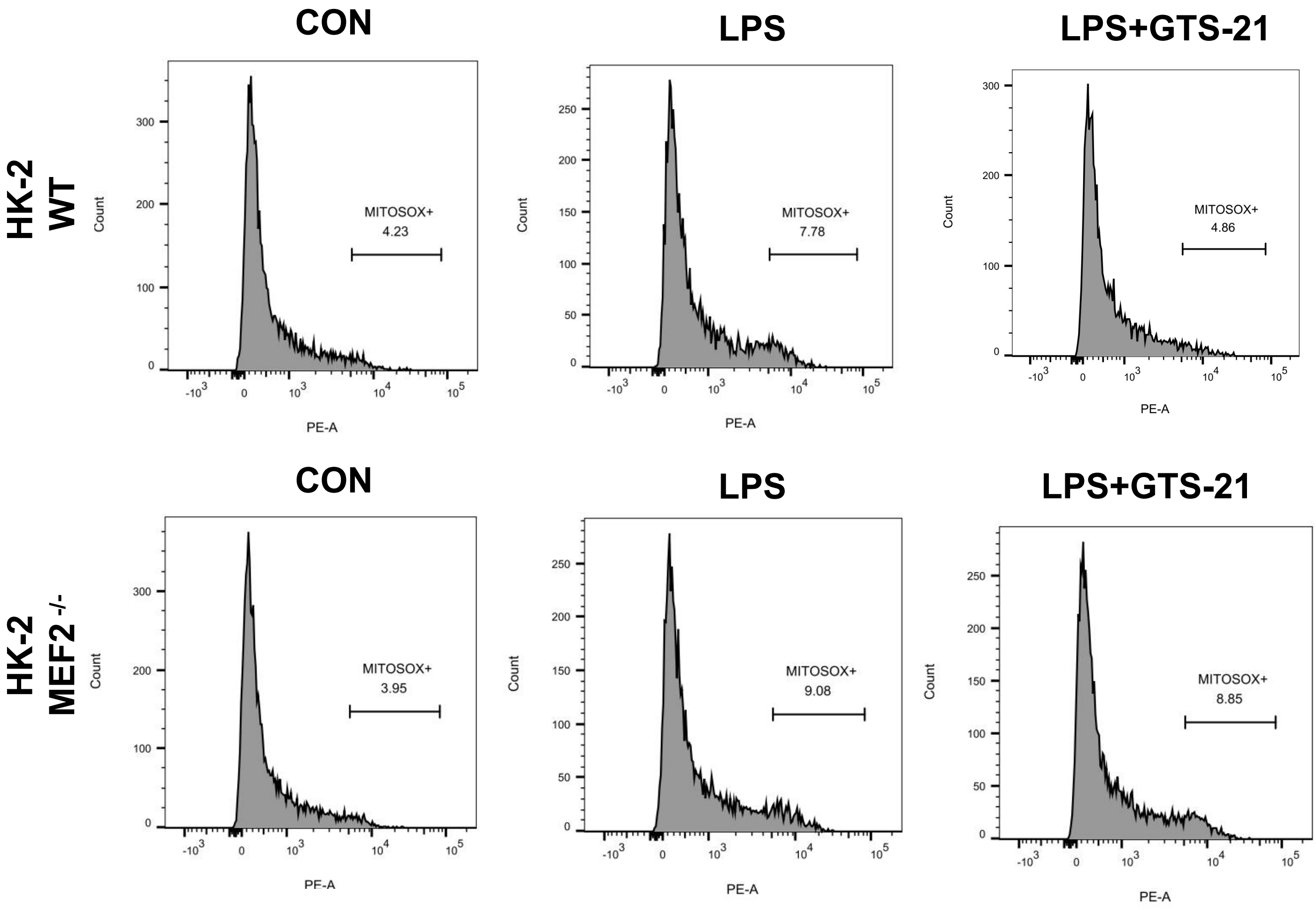

Fig.4. GTS-21 upregulates PGC-1α and HO-1 via MEF2 in HK-2 cells.
(C) Effects of α7nAChR agonists on mito-ROS levels in HK-2.

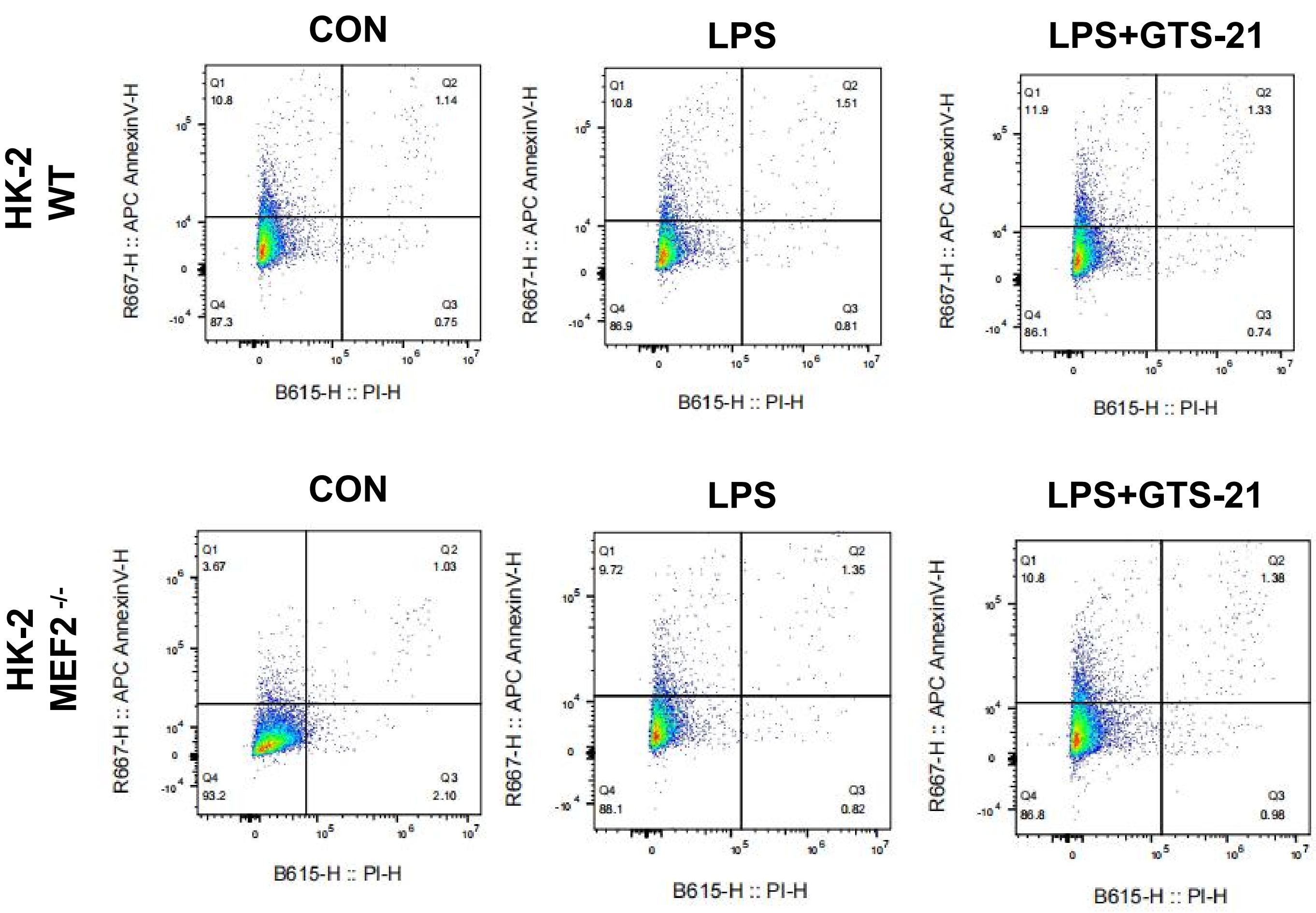

Fig.4. GTS-21 upregulates PGC-1α and HO-1 via MEF2 in HK-2 cells.
(D) Effects of α7nAChR agonists on apoptosis in HK-2.

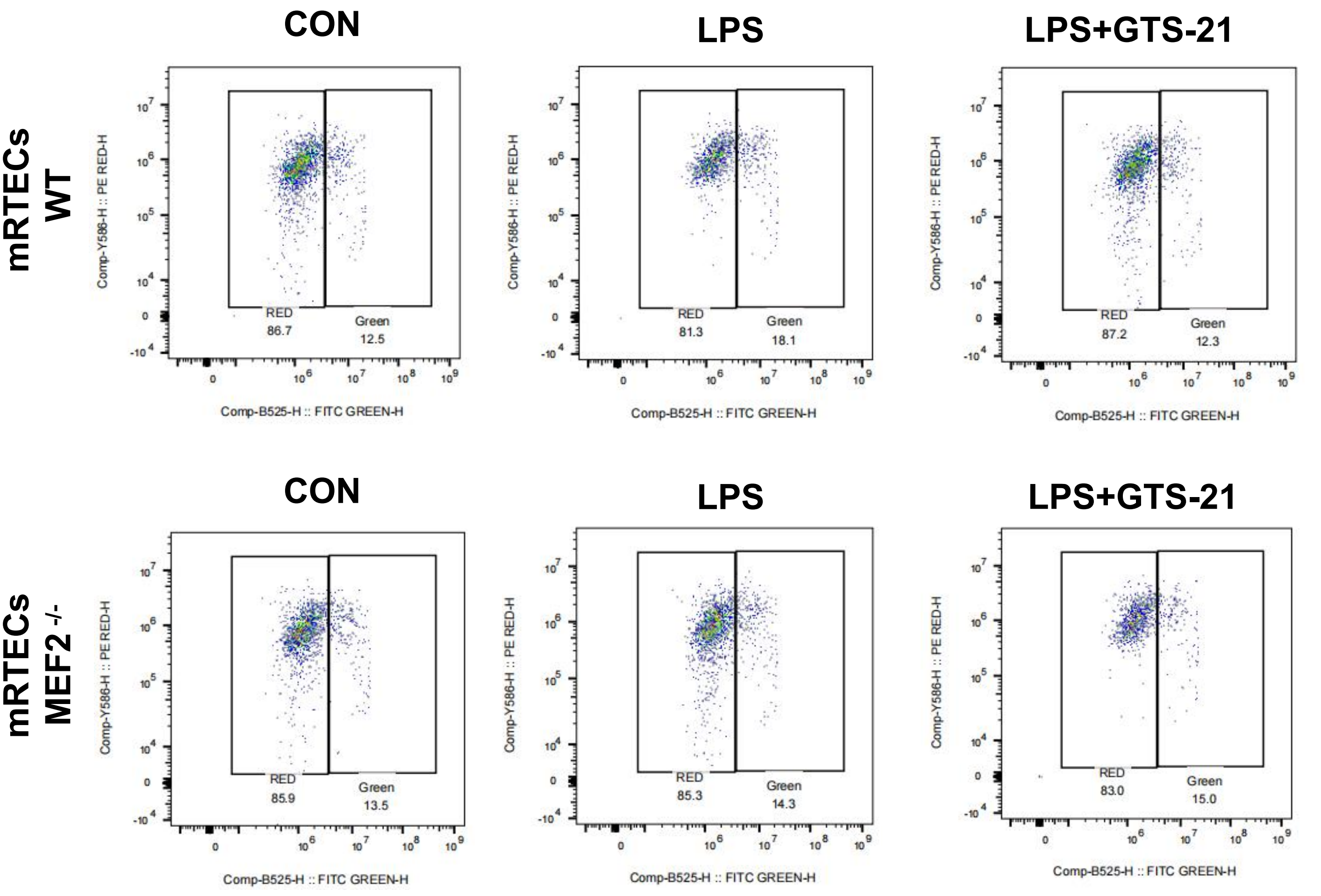

Fig.5. GTS-21 upregulates PGC-1α and HO-1 via MEF2 in mRTECs.
(B) Effects of α7nAChR agonists on MMP in mRTECs.

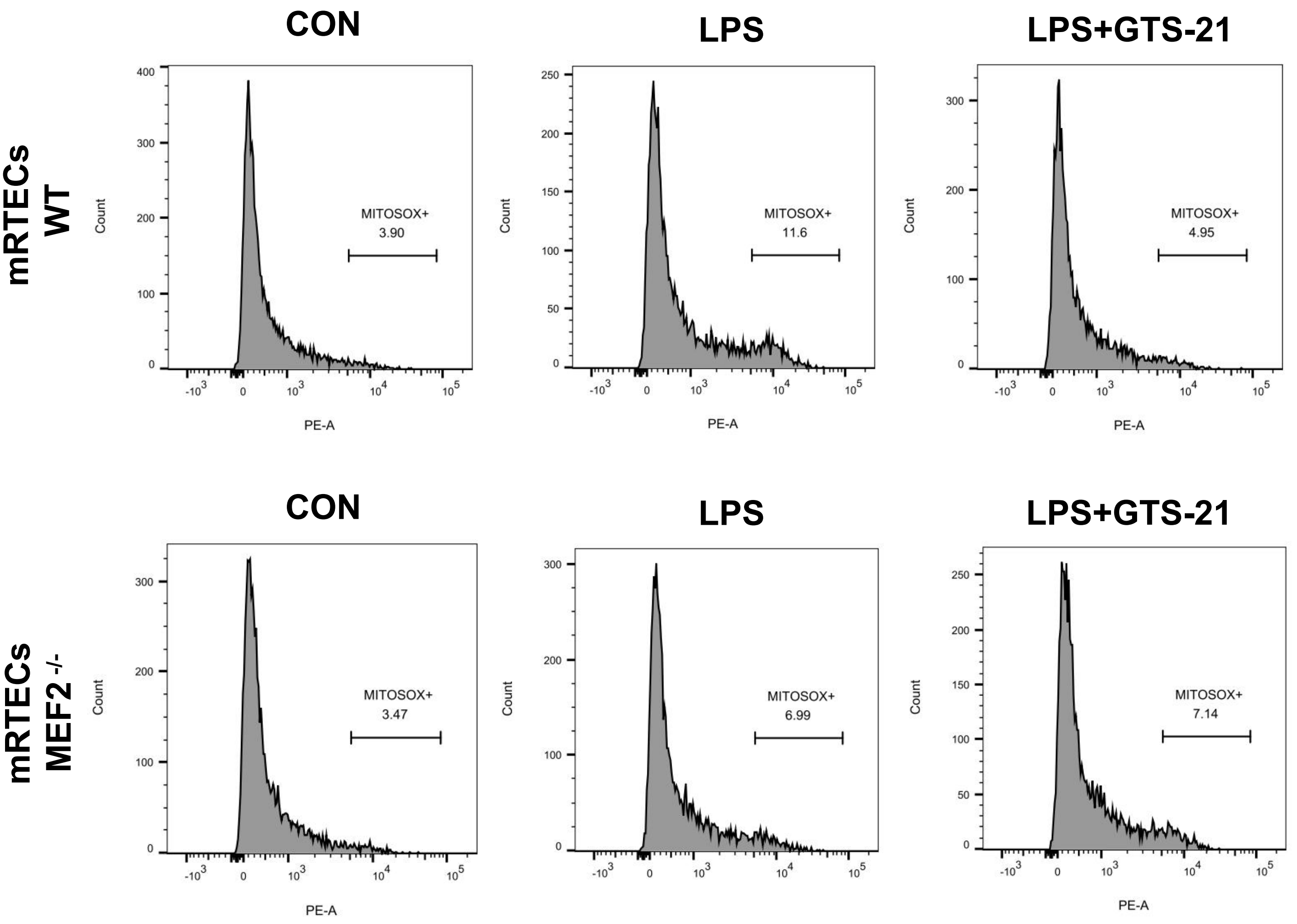

Fig.5. GTS-21 upregulates PGC-1α and HO-1 via MEF2 in mRTECs.
(C) Effects of α7nAChR agonists on mito-ROS levels in mRTECs.

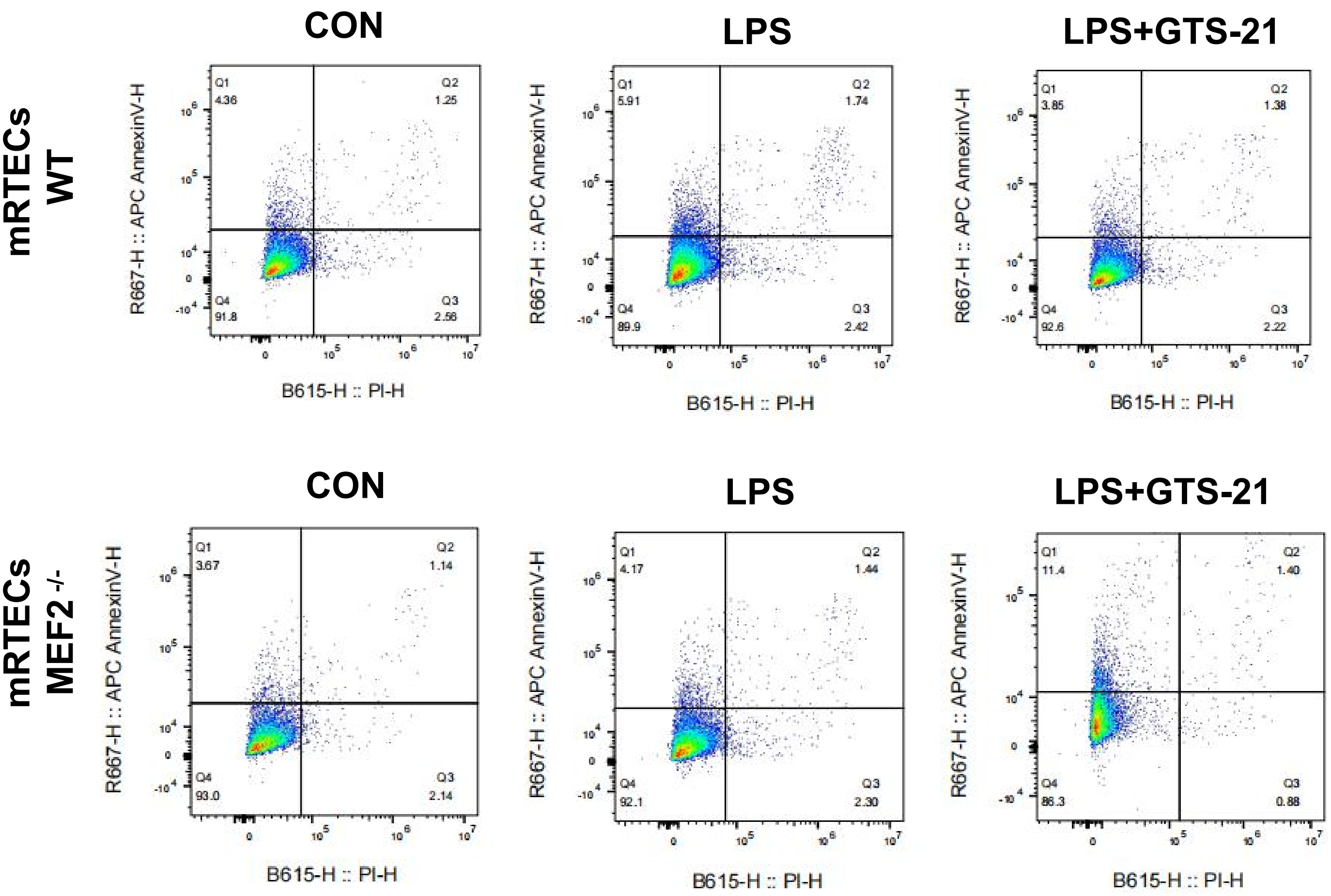

Fig.5. GTS-21 upregulates PGC-1α and HO-1 via MEF2 in mRTECs.
(D) Effects of α7nAChR agonists on apoptosis in mRTECs.

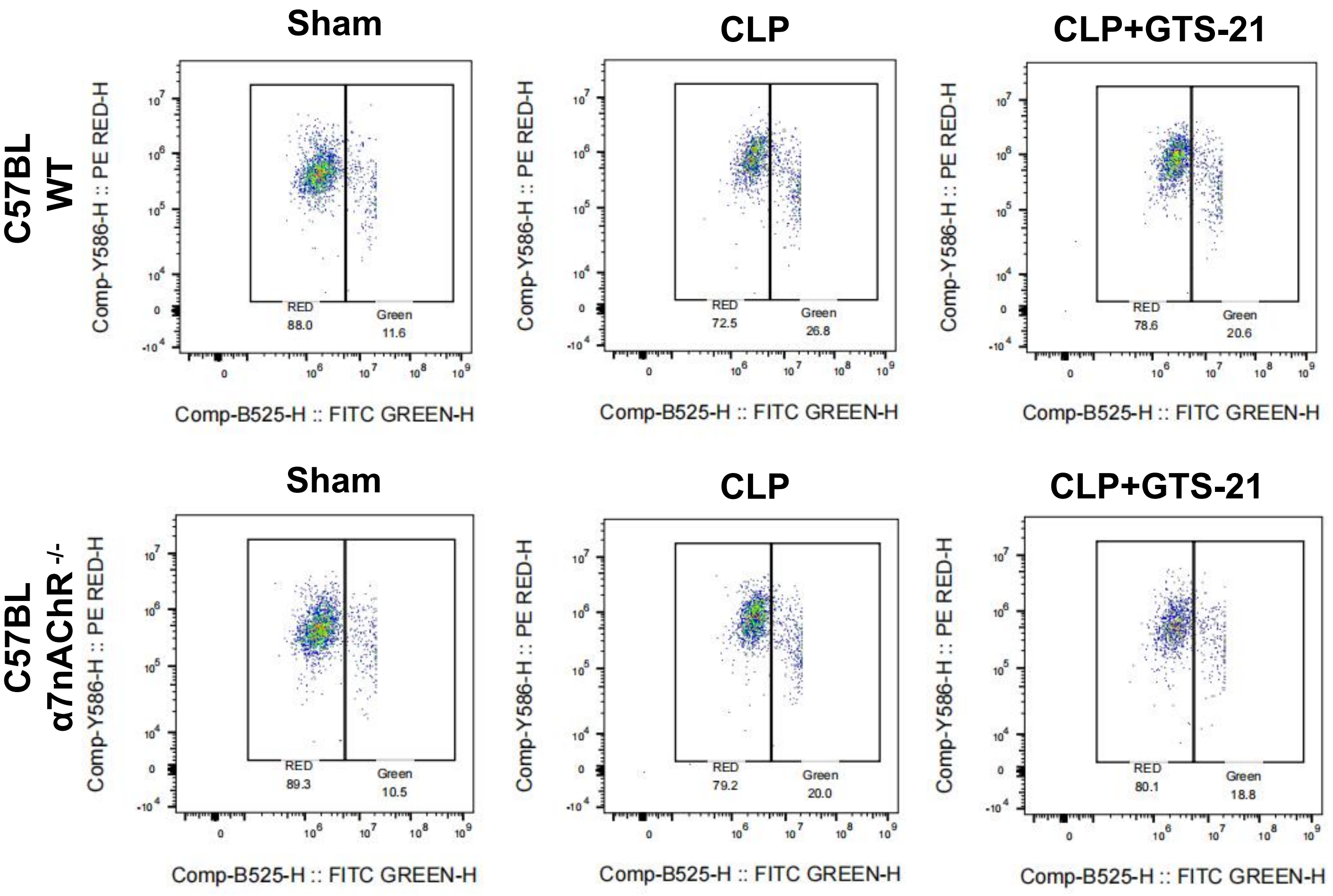

Fig.6. GTS-21 ameliorates mitochondrial damage and promotes mitochondrial biogenesis in CLP-induced mice.
(J) Effects of α7nAChR on MMP in CLP-induced mice.

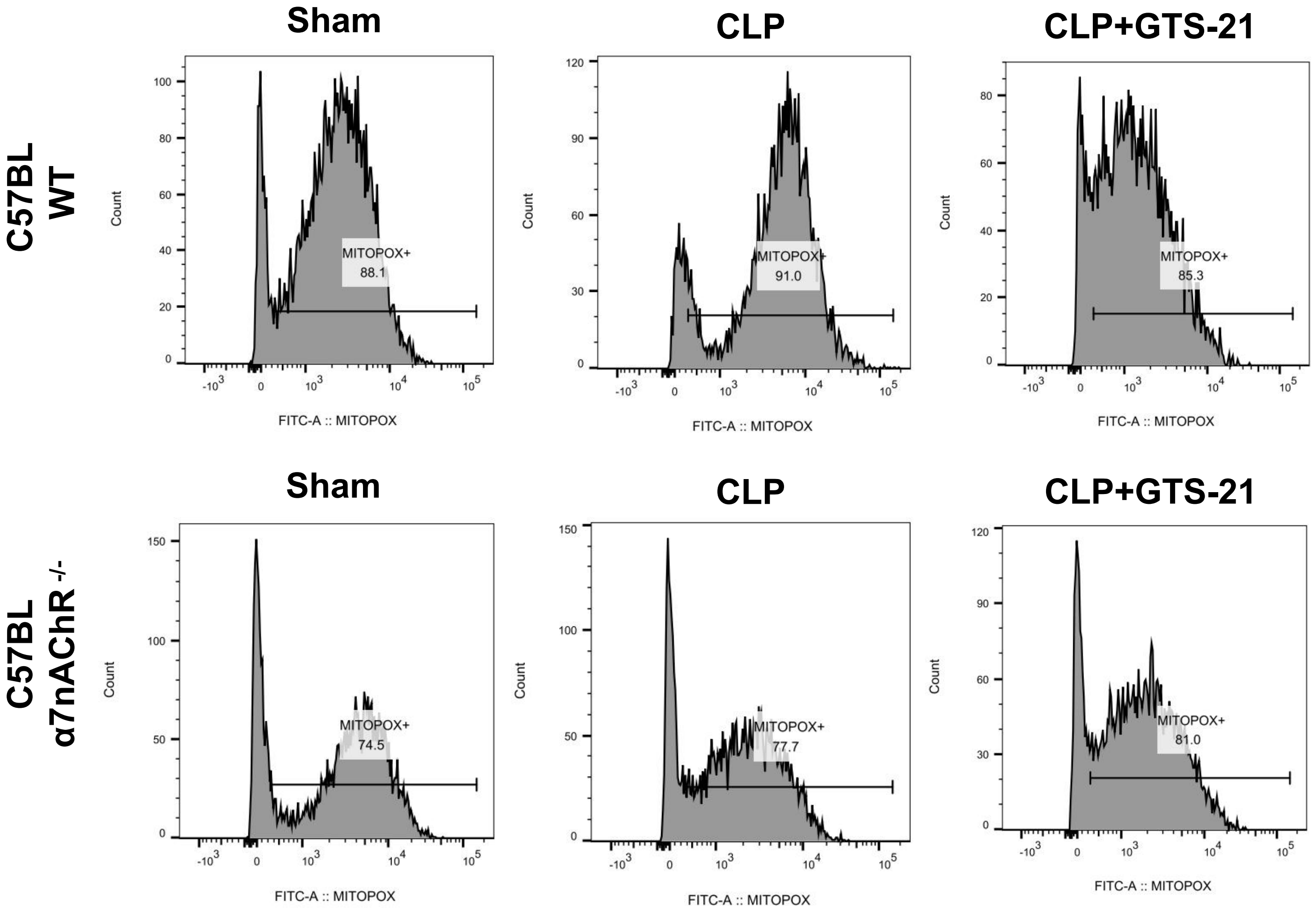

Fig.6. GTS-21 ameliorates mitochondrial damage and promotes mitochondrial biogenesis in CLP-induced mice.
(C) Effects of α7nAChR on mito-ROS levels in CLP-induced mice.

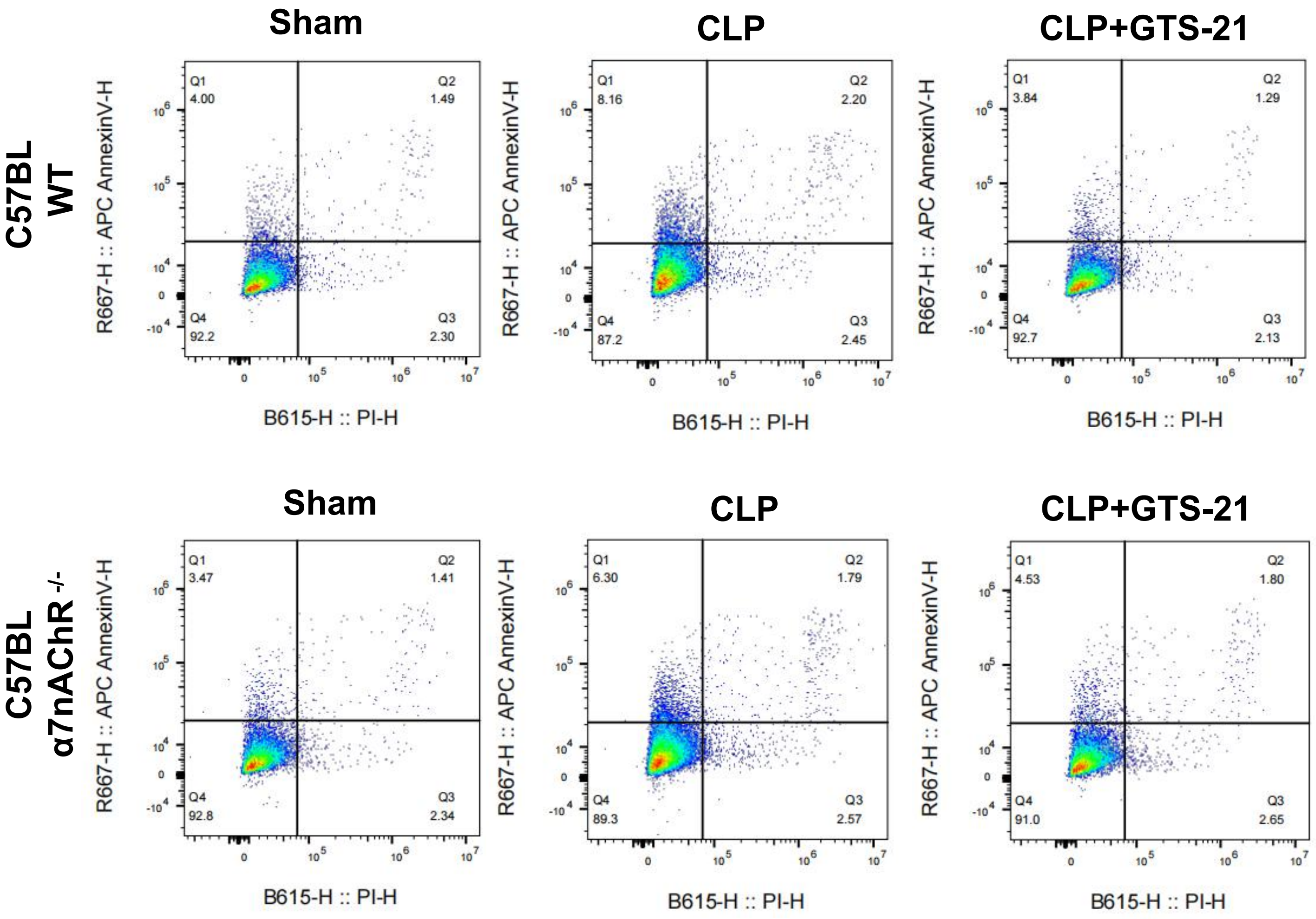

Fig.6. GTS-21 ameliorates mitochondrial damage and promotes mitochondrial biogenesis in CLP-induced mice.

(L) Effects of α7nAChR on apoptosis in CLP-induced mice.

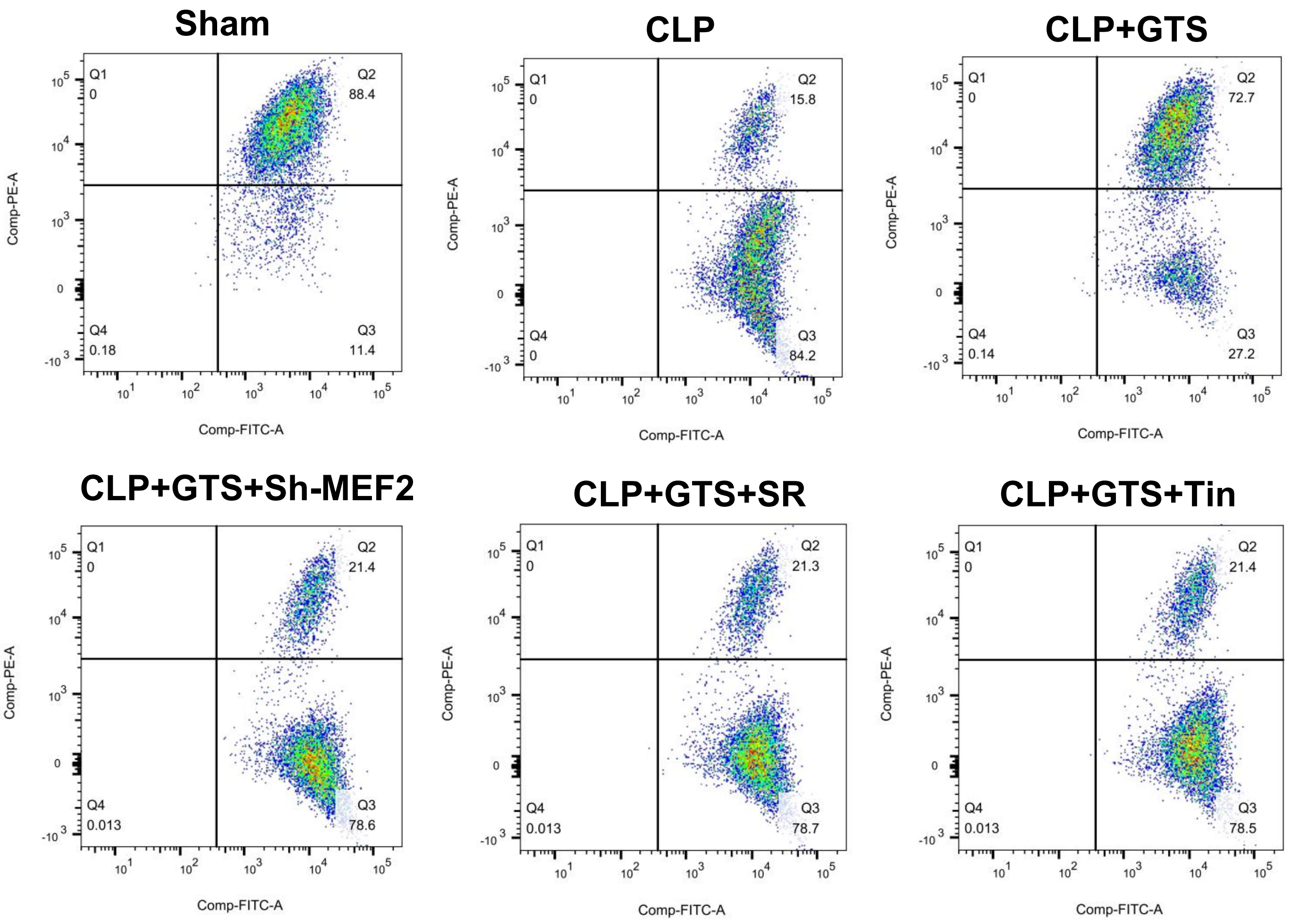

Fig.7. GTS-21 ameliorates CLP-induced injury in mice in a MEF2/PGC-1α/HO-1-dependent manner.

(I) Effects of MEF2 deficiency or inhibition of PGC-1α and HO-1 on MMP in CLP-induced mice.

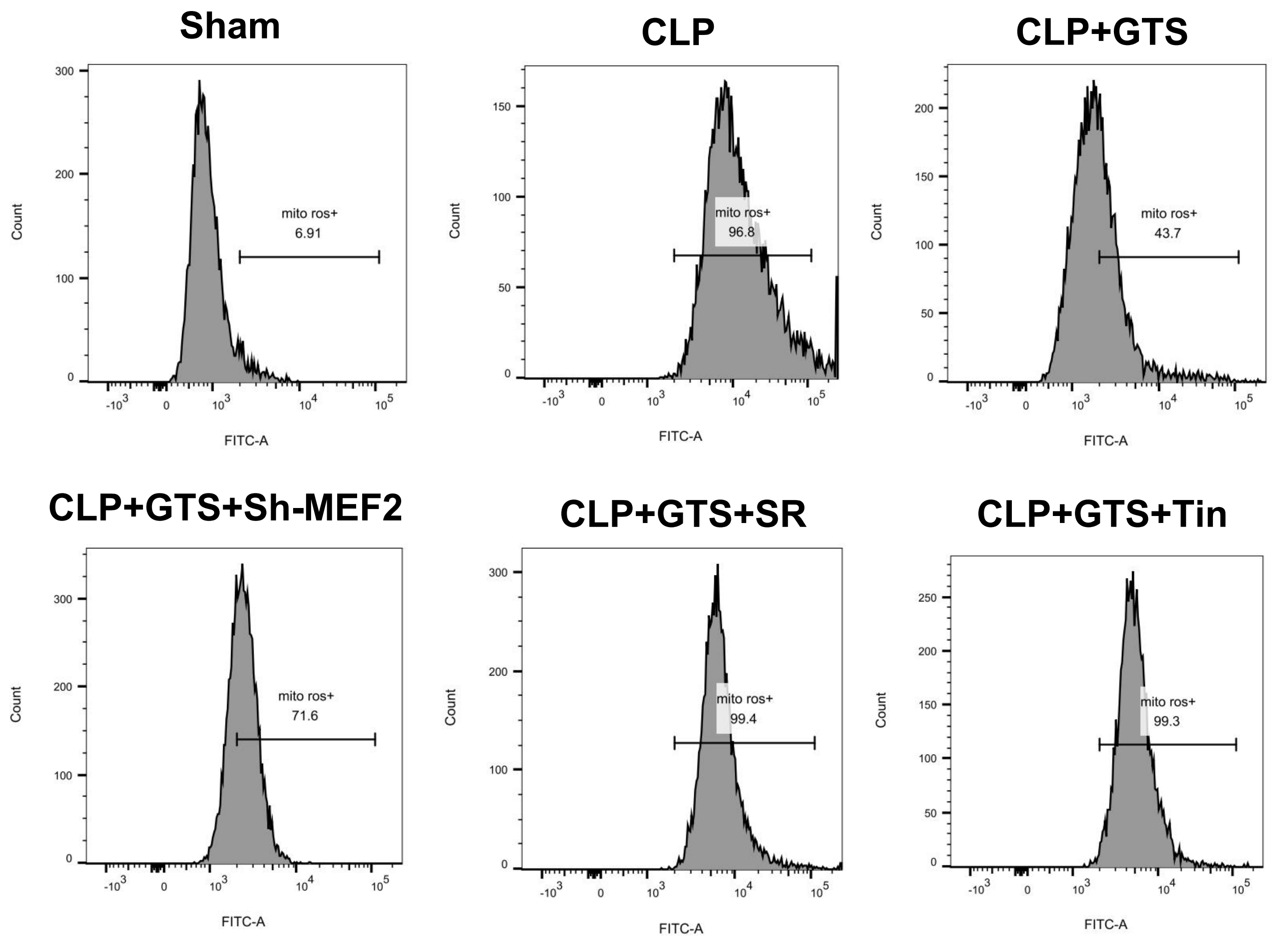

Fig.7. GTS-21 ameliorates CLP-induced injury in mice in a MEF2/PGC-1α/HO-1-dependent manner.

(C) Effects of MEF2 deficiency or inhibition of PGC-1α and HO-1 on mitochondrial ROS levels in CLP-induced mice.

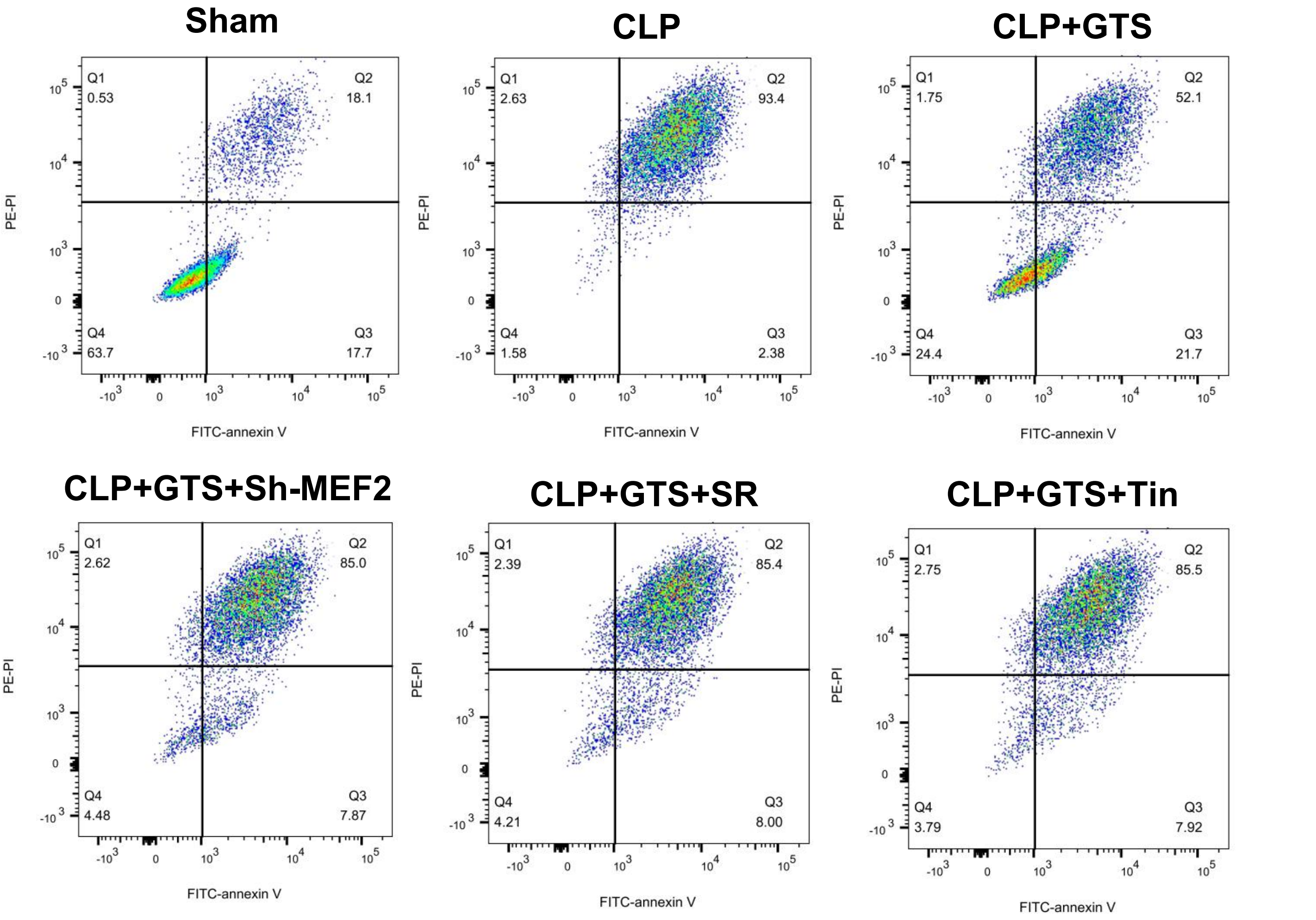

Fig.7. GTS-21 ameliorates CLP-induced injury in mice in a MEF2/PGC-1α/HO-1-dependent manner.

(I) Effects of MEF2 deficiency or inhibition of PGC-1α and HO-1 on apoptosis in CLP-induced mice.
