# Supplementary figures and images for: α7nAChR agonist GTS‐21 ameliorates sepsis‐induced acute kidney injury via MEF2/PGC‐1α/HO‐1 axis in mice
Source: Clin Transl Med. 2026 Jun 23;16(7):e70726. doi: 10.1002/ctm2.70726 (PMC13287963; doi:10.1002/ctm2.70726)

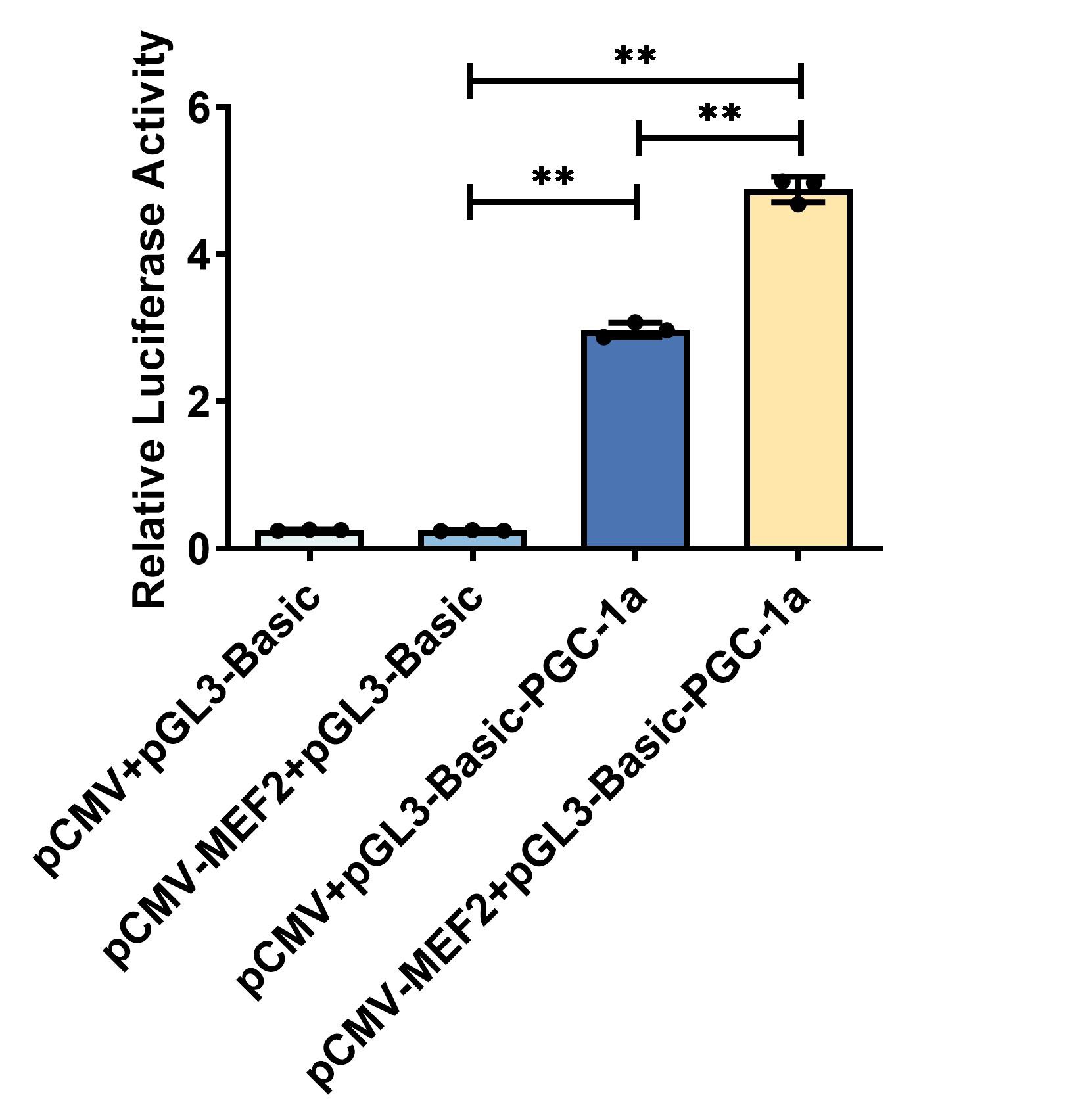

Supplement: Supplementary file 2 — Supporting Information [file CTM2-16-e70726-s002.tif]
